# DLGAP1 directs megakaryocytic growth and differentiation in an MPL dependent manner in hematopoietic cells

**DOI:** 10.1186/s40364-019-0165-z

**Published:** 2019-07-08

**Authors:** Boguslaw A. Kwiatkowski, Nicolas R. Burwick, Robert E. Richard

**Affiliations:** Seattle Institute for Biomedical and Clinical Research, VA Puget Sound Healthcare System, 1660 South Columbian Way, S-111-ONC, Seattle, WA 98108 USA

**Keywords:** DLGAP1, MPN, Mpl, Centrosomes, Megakaryocytes, Polyploidization

## Abstract

**Background:**

The MPL protein is a major regulator of megakaryopoiesis and platelet formation as well as stem cell regulation. Aberrant MPL and downstream Jak/STAT signaling results in the development of the Myeloproliferative Neoplasms (MPN). The pathogenetic and phenotypic features of the classical MPNs cannot be explained by the known mutations and genetic variants associated with the disease.

**Methods:**

In order to identify potential pathways involved in MPN development, we have performed a functional screen using retroviral insertional mutagenesis in cells dependent on MPL activation. We have used viral transduction and plasmid transfections to test the effects of candidate gene overexpression on growth and differentiation of megakaryocytic cells. The shRNA approach was used to test for the effects of candidate gene downregulation in cells. All effects were tested with candidate gene alone or in presence of hematopoietic relevant kinases in the growth medium. We assayed the candidate gene cellular localization in varying growth conditions by immunofluorescence. Flow Cytometry was used for testing of transduction efficiency and for sorting of positive cells.

**Results:**

We have identified the DLGAP1 gene, a member of the Scribble cell polarity complex, as one of the most prominent positive candidates. Analyses in hematopoietic cell lines revealed DLGAP1 centrosomal and cytoplasmic localization. The centrosomal localization of DLGAP1 was cell cycle dependent and hematopoietic relevant tyrosine kinases: Jak2, SRC and MAPK as well as the CDK1 kinase promoted DLGAP1 dissociation from centrosomes. DLGAP1 negatively affected the growth rate of MPL dependent hematopoietic cells and supported megakaryocytic cells polyploidization, which was correlated with its dissociation from centrosomes.

**Conclusions:**

Our data support the conclusion that DLGAP1 is a novel, potent factor in MPL signaling, affecting megakaryocytic growth and differentiation, relevant to be investigated further as a prominent candidate in MPN development.

**Electronic supplementary material:**

The online version of this article (10.1186/s40364-019-0165-z) contains supplementary material, which is available to authorized users.

## Background

The thrombopoietin receptor (myeloproliferative leukemia protein-MPL) activates the major signaling pathway that regulates megakaryocyte development and platelet production. Abnormal MPL signaling leads to the development of Myeloproliferative Neoplasms (MPN), a group of clonal stem cell disorders characterized by an overproduction of mature myeloid cells with a tendency to transform to acute myeloid leukemia (AML). The human Philadelphia Chromosome negative or “classic” MPN comprise three main subgroups: polycythemia vera (PV), essential thrombocytosis (ET), and primary myelofibrosis (PMF). Considerable progress has been achieved over the past decade in the understanding of MPN pathogenesis [1, 2]. Mutations in JAK2, MPL and in the calreticulin (CALR) genes have been identified as a central feature in most of these cases [3]. These three mutations result in direct or indirect dysregulation of JAK2 signaling with constitutive activation of cytokine dependent JAK-STAT/PI3K/AKT downstream signaling pathways. The direct activation of JAK2 results from the acquisition of a somatic JAK2 V617F mutation [4] or from somatic deletions, insertions and missense mutations, mostly in JAK2 exon 12 [5]. Mutations in MPL or CALR result in indirect dysregulation of JAK2 signaling [6, 7]. The CALR mutations are mutually exclusive with JAK2 and MPL, and in effect the CALR mutations account for up to 84% of JAK2 and MPL independent MPN. 15% of patients with ET and PMF do not have any of the standard driver mutations (JAK2, MPL and CALR). There is also a growing evidence indicating genetic events outside of JAK-STAT–activating mutations in MPN patients. Notably, mutational events preceding the acquisition of JAK2-V617F or MPL W515 L have been identified [8]. MPN related mutations beyond the JAK-STAT pathway include genes in epigenetic modifiers, such as TET2, DNMT3A, ASXL1, EZH2, IDH1, IDH2 [9], as well as genes belonging to other hematopoietic signaling pathways, including: LNK [10], CBL [11] as well as SOCS1, SOCS2 and SOCS3 [12].

Despite vast genetic data and functional in vitro and in vivo studies of the 3 described driver mutations the process by which MPNs transform to AML is not understood. Neither the drivers of clonal heterogeneity of MPN have not been established either [13]. New screening approaches could reveal genes that in cooperation with the MPN driver mutations may contribute to MPN initiation, clonal development and leukemic transformation. We used MSCV based insertional mutagenesis linked with expression of a dimerizable MPL construct to screen for factors that might give proliferative and/or survival advantage for cells dependent on MPL signaling. This method identified the *DLGAP1* gene, which product cooperates with MPL signaling in cell proliferation and polyploidization processes.

## Methods

### Vectors used

The MGIFMNOo, MSCV-based retroviral bicistronic construct, contained the Enhanced Green Fluorescent Protein-Internal Ribosomal Entry Site (EGFP-IRES) coding cassette [14] in MGIFMNOo, followed by MPL dimerization inducible construct coding for cytoplasmic domain of mouse MPL linked at its amino end to a 14-amino acid cytoplasmic membrane targeting myristylation domain and at its carboxy end to HA epitope tag. The MGIFMNOo construct contained also sequences coding for the Neomycin resistance gene and the p15 bacterial origin of replication, in its retroviral 3′ untraslated region creating the shuttle plasmid for genomic integration site rescue. The vector was provided by C. Anthony Blau, University of Washington.

The MFhuMIGNOo vector was cloned by replacing the sequences coding for cytoplasmic domain of mouse MPL in MFMIG vector (provided by C. Anthony Blau) with sequences coding for the cytoplasmic domain of human MPL, derived from pNF2hMpl (provided by C. Anthony Blau). The MFhuMIGNOo vector contains sequences coding for dimerization inducible construct based on human MPL upstream of IRES and coding sequences for the EGFP downstream of IRES.

The pEGFP-DLGAP1 vector was cloned by in frame ligation of full length DLGAP1 cDNA into Eco RI and Kpn I sites of the pEGFP-C1 vector (Clontech, Mountain View, CA). The full length DLGAP1 cDNA sequence with 5′ Eco RI site and 3′ Kpn I site was generated on 3197 bp full length cDNA sequence template of DLGAP1 from clone ID: 9020442 (MGC:168065 IMAGE:9020442) in pCR4-TOPO vector, acquired from Open Biosystems (Lafayette, CO). The PCR was performed using Phusion Polymerase system (New England Biolabs, Inc., Ipswich, MA) and primers as follows: forward primer: gcGAATTCcatgaaagggctatcaggc, reverse primer: gaGGTACCctgcgaggtggacaactaca.

The pEGFP-TrDLGAP1 vector was cloned by generating a 5′ end truncated cDNA sequence of DLGAP1 by using forward primer: gcGAATTCccaggatgcctacATGga instead and following the procedure of the pEGFP-DLGAP1 cloning as described above. The forward primer for pEGFP-TrDLGAP1 cloning carries sequence complementary to exon 5 region of DLGAP1 surrounding the first Methionine codon in this exon.

The constitutively active clone of CDK1: Flag-CDK1-AF, and the dominant negative clone of CDK1: Flag-CDK1-ND were a kind gift from Dr. Daniel Wu, VA Puget Sound Health Care System, Seattle.

### Cell cultures

Cell lines used include: 293 T [15], K562 (CCL-243, ATCC, Manassas, VA), UT7 [16], UT7/TPO [17], MO7e [18] and HEL [19]. The 293 T cells were grown in Dulbecco’s modified Eagle medium (DMEM (BioWhittaker, Walkersville, MD) with high glucose (4.5 g/liter) supplemented with 10% fetal bovine serum (FBS) (HyClone/Thermo Fisher Scientific Inc., Waltham, MA), with 50 U/ml penicillin and 50 mg/ml streptomycin. The K562 and HEL cell lines were grown in RPMI 1640 (HyClone/Thermo Fisher Scientific Inc., Waltham, MA) supplemented with 10% FBS (HyClone/Thermo Fisher Scientific Inc., Waltham, MA), with 50 U/ml penicillin and 50 mg/ml streptomycin. The UT7 and UT7/TPO were grown in IMDM medium (HyClone/Thermo Fisher Scientific Inc., Waltham, MA) supplemented with 10%, FBS (HyClone/Thermo Fisher Scientific Inc., Waltham, MA), with 50 U/ml penicillin and 50 mg/ml streptomycin. All cell lines were grown at 37 °C in an atmosphere containing 5% CO2.

When indicated, culture medium was supplemented with one of the following reagents: 10 pg/ml AP20187 - a dimerizer of proteins containing an F36 V domain, which is a modified FKBP-binding domain [33] (ARIAD Pharmaceuticals, Cambridge, MA), 1 μM Imatinib (Novartis, Basel, Switzerland), 10 pg/ml GMCSF, 50 ng/ml PEG-rhMGDF, 400 mg/ml Geneticin (Invitrogen, Carlsbad, CA), 50 μM AG490 (Cayman Chemical Co., Ann Arbor, MI [Cayman]), 2.5 μM SU6656 (Cayman), 20 μM U0126 (Cell Signaling Technology, Boston, MA), 100 ng/ml Nocodazole (SCBT), 40 nM PMA (SCBT), 200 μM GTP (SCBT), 50 μM Olomoucine (SCBT), 50 μM iso-Olomoucine (SCBT) and 4.5 μM or 9 μM RO3306 (SCBT).

Exposure times to the different reagents are indicated in the Results section.

### Viral transduction

Retroviral supernatants were generated by transient transfection of the packaging 293 T Phoenix gag-pol cells using Polyethylenimine, 25 kDa (Polysciences, Inc., Warrington, PA). Culture supernatants, containing packaged retroviral particles, were mixed with polybrene reagent (8 mg/ml) and used for infection of K562, UT7 and UT7/TPO cells. Cultures were exposed to viral supernatant for 4 h and then received fresh growth medium for 48 h, after that time period transduction efficiency was assayed and cells were supplied with drugs for appropriate selection.

### Flow cytometry

Cells were harvested and resuspended in PBS supplemented with 1% FBS. The EGFP expression was analyzed on either a FACSCAN or FACSCalibur (BD Biosciences, San Jose, CA, USA) using argon-ion laser excitation (488 nm). EGFP was detected using the FL1 parameter with emission filter: 530 ± 15 nm. Dead cells and debris were excluded by gating for intact cells using the forward and sideward scatter. Cells non expressing EGFP were used as control to detect autofluorescence. Data acquisition was carried out by analyzing minimum of 10 000 events/sample using CellQuest Software (BD Biosciences).

### Cloning and sequencing of retroviral integration sites

Genomic DNA was purified using QiaAmp DNA blood Mini Kit and QiaAmp DNA blood Midi Kit (QiaGen Inc., Valencia, CA) according to manufacturer manuals. The genomic DNA was cut with one of the following enzymes: Eco RI, Bam I, Hind III, Bgl II or Apo I, and liagted with T4 Ligase (New England Biolabs, Ipswich, MA) to recircularize the shuttle plasmids containing fragments of genomic DNA. In following steps the DNA was cut with Dpn I enzyme and elctroporated into DH10B cells. The shuttle plasmids with genomic fragments were purified from single bacterial clones using QiaGen Miniprep Kit and sequenced on ABI-PRISM sequencer using BigDye 3.1 chemistry (Applied Biosystmes, Inc., Foster City, CA) using an oligo primer derived from the sequence of MSCV LTR: 5′-GTTCGCTTCTCGCTTCTGTT-3′.

### Immunofluorescent microscopy

For immunofluorescent microscopy studies cells were fixed by adding 1.5 vol. of 3.7% formaldehyde in PBS at room temperature directly to cell culture for 12 min and to transferred to cytospin chambers fitted with Permafrost Plus slides (Fisher Sci., Pittsburgh, PA) and were spinned at 1600 RPM for 2 min in StatSpin Cytofuge (Iris Sample Processing, Westwood, MA). Cells on slides in cytospin chambers were then washed twice in 800 μl PBS and air dried for 16-24 h. For staining cells were permeabilized in 0.3% Triton X-100 in PBS for 10 min at room temperature following with blocking with 5% of appropriate normal serum and 1% BSA (Sigma, Fraction V, lipid-free) in PBS for 1 h at RT. Staining was performed by incubation with desired primary antibody (ies) in PBS containing 1% of appropriate normal serum and 1% BSA for 1 h at RT followed by O/N at 4C. After that cells were washed four times in PBS + 0.05% Tween 20. Incubation with secondary antibodies in PBS containing 1% of appropriate normal serum and 1% BSA was carried for 1.5 h at RT.

The secondary antibodies used were the following: 488 Alexa Fluor rabbit anti-Goat, 488 Alexa Fluor chicken anti-rabbit, 488 Alexa Fluor chicken anti-mouse, 568 Alexa Fluor goat anti-rabbit, 568 Alexa Fluor goat anti-mouse and 568 Alexa Fluor rabbit anti-goat (all from Invitrogen, Grand Island, NY). Cellular DNA was counterstained with 1 μM To-Pro3 Iodide, or 4′6-diamidino-2-phenylindole-2HCl (DAPI) for 15 min at RT, and cells were washed four times in PBS + 0.05% Tween 20. Finally, slides were air dried and coverslips were mounted using one drop of VectaShield solution (Vector Laboratories, Inc., Burlingame, CA).

All imaging was performed on Leica TCS-SP 1 Confocal Laser Scanning System attached to the Leica DM-R Upright Fluorescent microscope (Leica Microsystems Inc., Heidelberg, Germany) or on Nikon Eclipse E-80 microscope fitted with Filters for: DAPI, FITC, TRITC, Cy5 and a camera: QImaging Retiga 2000R (original magnification × 400).

### Antibodies

Primary antibodies used in Immunostaining and Western blots included: anti-APC (sc-896, Santa Cruz Biotechnology, Santa Cruz CA [SCBT]), anti-DLGAP1 (sc-25662, SCBT), anti-DLGAP1 (sc-12219, SCBT), anti-DLGAP1 (75–236, NeuroMab/ Antibodies Inc., Davis, CA), anti-PCM-1 (CS #5213, Cell Signaling Technology, Boston, MA), anti-α-Tubulin (13–8000 Zymed/Invitogen, San Francisco, CA), anti-γ-Tubulin (sc-17787, SCBT).

### Sequence analysis software

DNA and protein sequence analysis were performed using the Vector NTI software (Invitrogen). The nucleic acid and protein sequence data library searches were performed using Internet based: The Basic Local Alignment Search Tool (BLAST) (National Center for Biotechnology Information) and the University of California Santa Cruz Genome Browser search engines.

## Results

### Retroviral insertions into the fourth intron of DLGAP1 result in MPL dependent cell proliferation

**Fig. 1:**
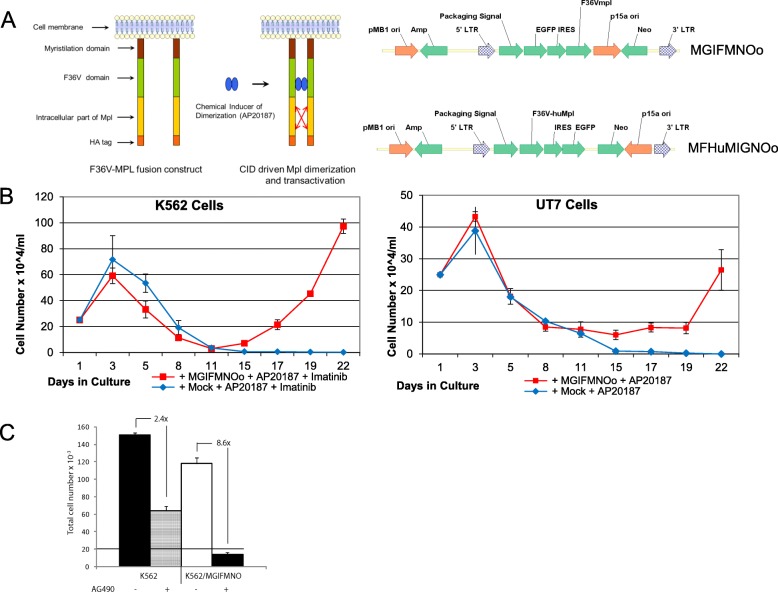
Insertional mutagenesis screening system for MPLsignaling cooperating gene factors. **a** Left panel represents schematic of the inducible MPL signaling system comprising the F36 V-MPL fusion construct that upon addition of the AP20187 drug leads to the MPL domain dimerization and transactivation followed by downstream MPL signaling processes. Right panel shows schematics of the MGIFMNOo and MFHuMIGNOo constructs used in functional screens in K562 and UT7/TPO cell lines. **b** Line graphs of K562 and UT7 cells selection on AP20187 after MGIFMNOo transduction. **c** Bar graph of 4 days treatment of plain and MPL driven K562 cells with AG490. The horizontal line at 20 × 10^3 represents the number of seeded cells

We used the Murine Stem Cell Virus (MSCV) based retroviral construct containing additional sequences of an artificial signaling protein based on the MPL receptor [20] to identify genes that cooperate with MPL signaling. The signaling of this construct is achieved by supplying a drug (AP20187) that binds the FK506 domain and dimerizes the fusion MPL protein [21]. Dimerization results in activation of the MPL-JAK2 pathway (Fig. 1a). In previous studies this system successfully directed a drug dependent expansion of blood progenitors [14, 22] in a Jak2 dependent manner [23]. We added a shuttle plasmid [24, 25], into the 3′ untranslated region of the retroviral sequences, so that insertion sites could be identified (Fig. 1a). This shuttle plasmid (NO) contains the p15A bacterial origin of replication and the Neo gene with Tn5 promoter allowing for a non-biased recovery of insertion sites in bacteria. A modified version of that construct: MFhuMIGNO (Fig. 1a) used the human MPL sequence to obtain a signaling protein fully compatible with human hematopoietic cell lines used in our screenings. The screening construct can interrupt gene structure through insertion and can provide promoter and enhancer activities through its long terminal repeats (LTRs) [14].

We utilized the human hematopoietic cell lines K562, UT7, and UT7/TPO in our screening. We intentionally blocked BCR-ABL signaling with Imatinib in K562 cells, which are dependent on it for survival, and substituted it with inducible MPL signaling through the retroviral delivery of one of our MPL signaling constructs: MGIFMNO or MFHuMIGNO in the presence of the AP20187 drug in the growth medium. The UT7 and UT7/TPO cell lines require cytokines supplementation, either GM-CSF or TPO, for long term growth. After selection we identified recurrent retroviral insertion sites (RISs). This allowed for the identification of secondary, cooperating factors supporting MPL driven survival and proliferation. Imatinib resistant clones have arisen in culture at a rate < 0.1% (Fig. 1b). The selected, GFP positive cells, were dependent on signaling through JAK2 as evidenced by complete sensitivity to the JAK2 inhibitor AG490 (Additional file 1). In our system four-day treatment of MPL dependent K562 cells with AG490 resulted in 8.6-fold growth retardation, while the same treatment of plain K562 cells resulted in only 2.4-fold growth inhibition (Fig. 1c). Previous studies in K562 cells indicated that Imatinib treated cells differentiate into red cells and die [26]. Another important observation indicated that only a minority of MGIFMNO containing cells expand. In separate experiments in which sorted, uniformly GFP+ cultures were selected with AP20187 and Imatinib, only 3% of the cells survived the selection process and gave rise to the subsequent culture (Additional file 1). This indicates a secondary feature to the surviving cells that affords a selective advantage.

**Fig. 2:**
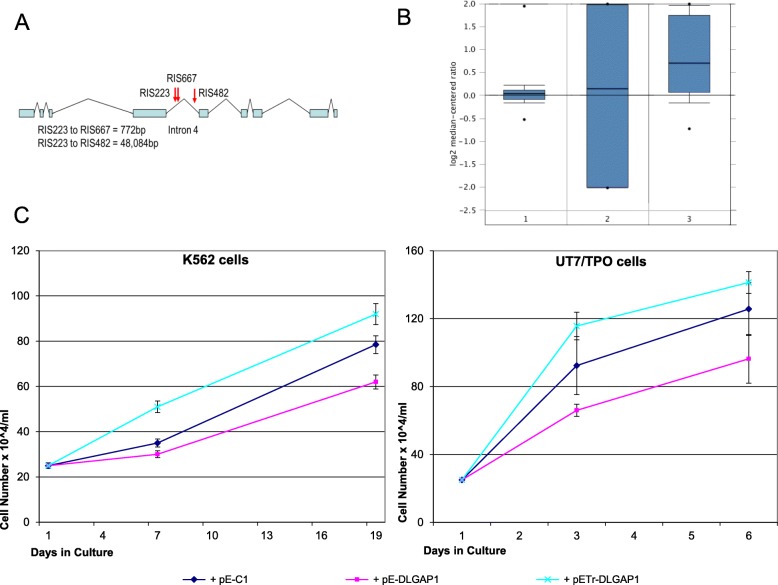
DLGAP1: gene structure, levels in CML and effects on cell growth. **a** Schematic of DLGAP1 gene structure with blue bars representing exons and red arrows indicating 3 independent RISs localization. Distances between the rescued RISs are given below. **b** Bar graph of relative DLGAP1 mRNA levels in different phases of CML from FHCRC patient database: 1 chronic phase, 2 accelerated phase, 3 blast crisis (modified from Oncomine.org). **c** Effect of DLGAP1 isoforms on growth of MPL driven cell lines. Left panel: line graph of the growth of K562 cells driven by inducible MPL construct and overexpressing the full length and truncated isoform of DLGAP1. Right panel: UT7/TPO cells supplemented with rhMGDF and overexpressing the full length DLGAP1 from pE-DLGAP1 vector and its truncated form from the pE-Tr-DLGAP1 vector

We performed 36 independent transductions with 30,000 independent insertion events each, with an estimated total of ~ 1.5 × 10^6^ independent insertions in the initial non-selected cell population. After selection with Imatinib and AP20187, 668 retroviral insertion sites (RIS) were recovered that represent 203 independent insertion events. Within each independent transduction, a small number of RISs were identified suggesting selection of insertion events that result in a proliferative advantage. Such restriction was not observed in an independent experiment in K562 cells, in which the cells were not switched to MPL dependent growth (data not shown). Analyses of the 203 independent sites demonstrate skewing to a preponderance of insertions near genes that regulate apoptosis or are involved in cell signaling (Additional file 2). Among the multiple close proximity RISs in our screen three insertions occur in the fourth intron of the discs-large associated protein 1 (*DLGAP1*) gene (Fig. 2a), a member of the Discs-large/ Scribble/Lethal Giant Larvae pathway. This pathway has been implicated in controlling cell proliferation and polarity [27, 28].

### DLGAP1 expression was upregulated in human myeloid malignancies in published data sets from high throughput gene expression studies

Since K562 cells arose from a CML associated erythroleukemia, we examined relative levels of DLGAP1 mRNA in a database at the FHCRC of patient samples in all the phases of CML as well as normal CD34 cells. *Dlgap1* mRNA expression increases 10–100-fold in the blast phase relative to chronic phase CML expression levels (Fig. 2b). In a follow-up examination we queried the Oncomine Platform (www.oncomine.org) and we found data from other studies indicating a statistically significantly increased expression of DLGAP1 in myeloid malignancies in humans (Additional file 3), while the copy number of DLGAP1 in myeloid malignancies was not noticeably changed based on the available datasets (Additional file 3).

### Overexpression of DLGAP1 isoforms affects the growth rate of hematopoietic cells

The localization of integration sites in our DLGAP1 RIS candidate clones suggested that the retroviral insertion could upregulate at least one truncated DLGAP1 isoform. We hypothesized that the expressed isoform is amino end truncated at the 4th intron, removing five 14 amino acid repeat domains but retaining the carboxy end GH1 GKAP homology domain. The insertion could also upregulate the full-length form of DLGAP1. To test the possible effect of these isoforms on the growth of MPL driven K562 and UT7/TPO cells we cloned the respective cDNAs into a pEGFP-C1 vector as carboxy end fusions with the EGFP protein. Overexpression of the full length DLGAP1 had minimal effect on plain K562 cells (data not shown), but it significantly retarded the proliferation of K562 cells switched to MPL signaling (Fig. 2c). We then tested the effects of overexpression of the truncated isoform in parallel with the full length DLGAP1 in the MPL dependent K562 and UT7/TPO cells. In each case the overexpression of full length DLGAP1 had a retarding effect on growth of the MPL dependent cells (Fig. 2c). Interestingly, the amino end truncated isoform had an opposite effect, increasing the proliferation rate in each case of the tested MPL dependent cells (Fig. 2c).

### DLGAP1 colocalizes with centrosomal markers and presents a cell cycle dependent centrosomal satellite pattern independent from PCM1 satellites

**Fig. 3:**
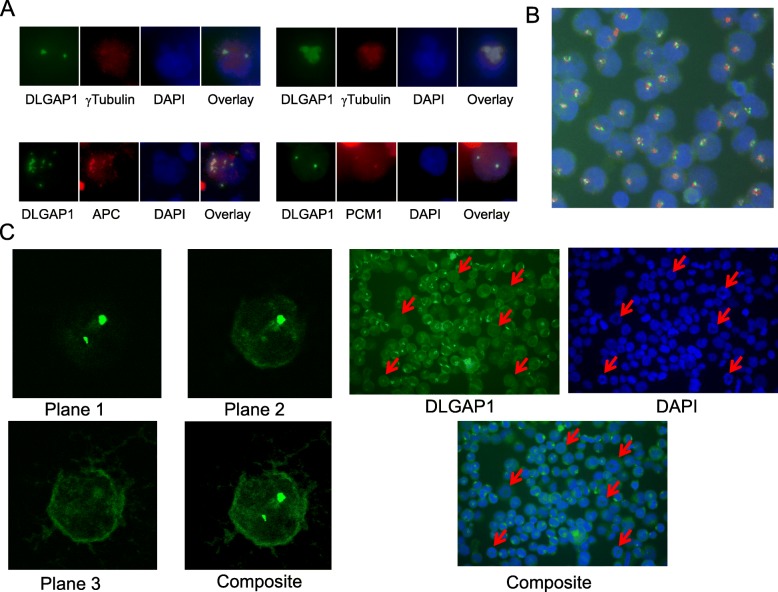
DLGAP1 localization in hematopoietic cells. **a** Immunofluorescent microscopy of DLGAP1 colocalization with centrosomal markers: γ-Tubulin, APC and PCM1. **b** Native DLGAP1 in M-O7e cells. DLGAP1 was stain green with specific antibody and DNA was stained blue with DAPI. **c** Left panel: confocal microscopy of native DLGAP1 in K562 cells. Anti-DLGAP1 antibody was used for staining DLGAP1 in green. Three single plane and one composite image are shown. Right panel: Immunofluorescent microscopy of native DLGAP1 in UT7-TPO cells. DLGAP1 is stained green with specific antibody and DNA is stained blue with DAPI. Arrows point to mitotic cells

Immunofluorescent studies unexpectedly revealed that the full length DLGAP1 colocalized with the centrosomal markers gamma-tubulin and APC, and occupied areas close to PCM1 localization (Fig. 3a). Such colocalization was not observed with the truncated isoforms of DLGAP1 (data not shown). DLGAP1 colocalized with gamma-Tubulin (centrosomal specific) in centrosomes and with APC in centrosomes and in its cytoplasmic speckles (Fig. 3a). Detailed Immunofluorescent analysis of the native DLGAP1 indicated that in centrosomal regions it is organized in small granules resembling centriolar satellites, as shown in the childhood acute megakaryoblastic leukemia cell line M-O7e (Fig. 3b). The DLGAP1 satellites were independent from the PCM1 centriolar satellites. DLGAP1 forms a punctate circular pattern around that of PCM1 satellites with varying number and size of the punctate speckles (Fig. 3b). Detailed confocal analyses of native DLGAP1 in K562 cells indicated that besides its centrosomal localization DLGAP1 has a discrete cytoplasmic presence and a localized concentration close to the cell membrane (Fig. 3c, left panel).

Immunofluorescent staining of native DLGAP1 in UT7-TPO cells revealed that the characteristic speckled centrosomal satellite pattern dispersed in mitotic cells while the discrete cytoplasmic and cell peripheral DLGAP1 patterns were not affected in those cells. Importantly, the DLGAP1 centrosomal speckles were not observed in any of the metaphase plate presenting cells (Fig. 3c, right panel). The same DLGAP1 patterns in non-mitotic and mitotic cell was observed in K562 and HEL and UT7 cell lines (data not shown).

### Treatment of cells with tyrosine kinase inhibitors: AG490, SU6656 and U0126 (inhibitors of JAK2, SRC and MAPK kinase activity respectively) results in dissociation of DLGAP1 from centrosomes

**Fig. 4:**
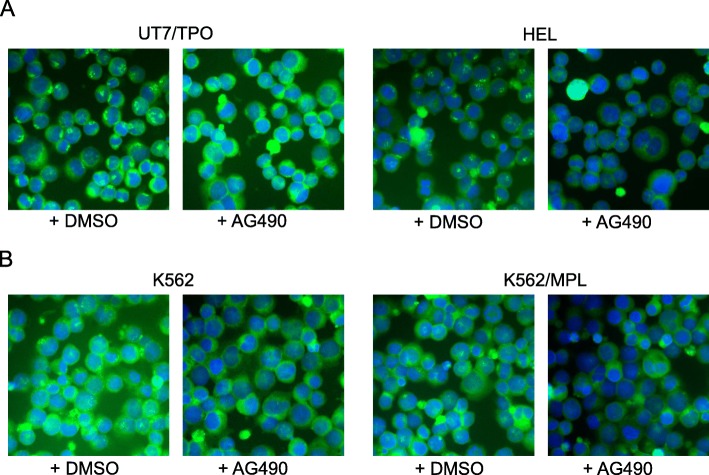
Effect of JAK2 tyrosine kinase inhibitor (+AG490) on DLGAP1 in hematopoietic cells. **a** Native DLGAP1 in UT7/TPO and HEL cells untreated (+DMSO) or treated with JAK2 tyrosine kinase inhibitor (+AG490). DLGAP1 was stained green with specific antibody and DNA was stained blue with DAPI. **b** Native DLGAP1 in plain K562 and in K562 cells driven by MPL signaling cells untreated (+DMSO) or treated with Jak2 tyrosine kinase inhibitor (+AG490). DLGAP1 was stained green with specific antibody and DNA was stained blue with DAPI

In silico analyses (PhosphoMotif Finder at Human Protein Reference Database (http://hprd.org/) indicated consensus phosphorylation sites in DLGAP1 protein sequence for hematopoietic relevant Tyrosine kinases including Jak2, SHP 1 and SRC (Additional file 4). The analyses also indicated multiple consensus sequences for MAPK and for CDK1 and CDK5 phosphorylation on Serine residues (Additional file 4). We investigated the potential influence of hematopoietic relevant Tyrosine kinases: JAK2, SRC and MAPK on DLGAP1 in cells. We treated hematopoietic cell lines with the relevant Tyrosine kinase inhibitors and analyzed DLGAP1 by immunofluorescence. The JAK2 inhibitor AG490 was added to cell cultures of UT7/TPO (Fig. 4a), HEL (Fig. 4a), K562 (Fig. 4b) and K562 driven by inducible MPL (Fig. 4B). JAK2 inhibition resulted in a complete disappearance of the characteristic native DLGAP1 centrosomal pattern by immunofluorescence (Fig. 4a-b).

To compare the effect of other hematopoietic relevant kinase inhibitors on DLGAP1 we performed immunostaining of endogenous DLGAP1 in UT7/TPO cells after treatment of the cells with AG490, SU6656 or U0126. In each case the treatment resulted in disappearance of the characteristic, punctate DLGAP1 centrosomal speckles (Additional file 5).

Interestingly, the respective kinase inhibitor treatments, which disrupted the centrosomal localized structures of DLGAP1, did not affect the centrosomal localization and architecture of the PCM1 satellites (Additional file 5). Such diversified effect on DLGAP1 and PCM1 centrosomal satellites was observed in the case of each inhibitor: AG490, SU6656 and U0126 in all four tested cell lines (K562, MPL dependent K562, HEL and UT7/TPO) (Additional file 6).

### Overexpression of DLGAP1 in hematopoietic cells results in increased frequency of polyploid cells and potentiates the polyploidization processes driven by SU6656

**Fig. 5:**
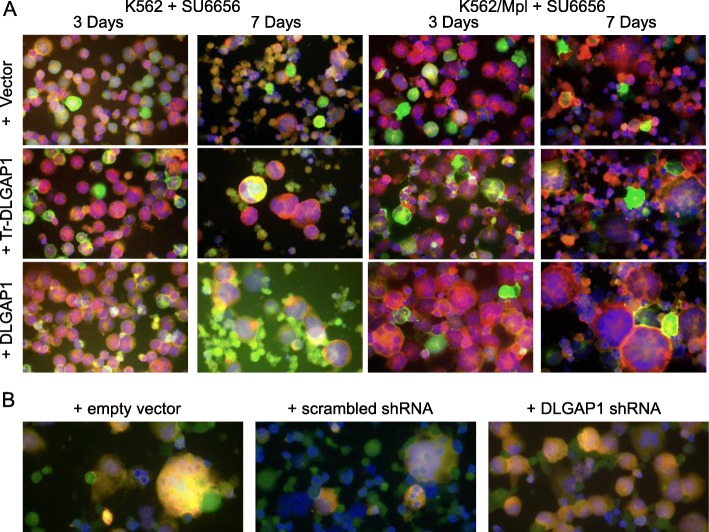
DLGAP1 effects on megakaryocytic polyploidization processes. **a** Immunofluorescent microscopy of plain K562 and K562 cells driven by MPL signaling overexpressing GFP-DLGAP1 and GFP-Tr-DLGAP1 treated for 3 and 7 days with SU6656. GFP is fluorescing green, alpha-Tubulin was stained red specific antibody and DNA was stained blue with DAPI. **b** Immunofluorescent microscopy of HEL cells driven into megakaryocytic differentiation by 6 days treatment with Nocodazole and PMA. Cells were grown in medium supplemented with empty vector, scrambled shRNA and specific DLGAP1 shRNA as indicated. Native DLGAP1 was stained green with anti-DLGAP1 antibody, shRNA vectors expressed RFP fluorescing red and DNA was stained blue with DAPI

To assess the possible involvement of DLGAP1 in hematopoietic cell differentiation we overexpressed the GFP-DLGAP1 fusion protein and its amino truncated isoform in K562 and in MPL dependent K562 cells, and then treated with the SRC inhibitor SU6656. This drug is a potent inducer of polyploidization and megakaryocytic differentiation in hematopoietic cells. We observed only a minimal effect on cells ploidy in the plain K562 after a 3-day treatment with the inhibitor. The full lengths DLGAP1 expressing cells indicated a slightly increased ploidy, while this effect was less pronounced in the amino truncated DLGAP1 expressing cells and only sporadic cells have shown polyploidization in the empty vector transformed cells (Fig. 5a). These effects were better pronounced after 7-day treatment with SU6656 with markedly higher ploidy cells emerging in the full length DLGAP1 expressing K562 cells (Fig. 5a). Such cells were less frequent in the amino truncated DLGAP1 expressing cells, and only a minor effect was observed in the empty vector transformed K562 cells (Fig. 5a). Interestingly, we observed a more dramatic effect in the MPL dependent K562 cells where a three-day treatment with SU6656 resulted in significant levels of polyploidization in the majority of the DLGAP1 expressing cells. Less frequent higher ploidy cells appeared in the amino truncated DLGAP1 expressing population, and only a low ploidy cells were observed in the empty vector carrying cells (Fig. 5a). A seven-day treatment with SU6656 of these cell populations resulted in a truly dramatic rate of polyploidization of the full length DLGAP1 overexpressing cells, showing frequent cells in very advanced ploidy state in the culture (Fig. 5a). Overexpression of the truncated version of DLGAP1 also resulted in a strong polyploidization but with less frequent cells of very advanced ploidy in the culture (Fig. 5a). Parallel treatment of the same cells carrying the empty vector yielded only low ploidy cells (Fig. 5a).

### Downregulation of DLGAP1 expression by specific shRNA blocks the megakaryocytic differentiation of hematopoietic cells driven by simultaneous treatment with Nocodazole and PMA

We have tested the possible role of DLGAP1 in megakaryocytic differentiation by applying the DLGAP1 specific shRNA to a culture of HEL cells that we treated in parallel with Nocodazole and PMA, a potent combination of factors inducing megakaryocytic differentiation in model cell systems. After 6 days in culture the DLGAP1 shRNA treated UT7/TPO culture demonstrated only negligible increased cell diameters, consistent with early stages of megakaryocytic polyploidization (Fig. 5b). Importantly, no cells in advanced stages of megakaryocytic differentiation and high ploidy were observed in the DLGAP1 shRNA treated populations, when such cells were frequent in the empty vector supplied culture (Fig. 5b). The control, scrambled shRNA culture indicated cells undergoing advanced megakaryocytic differentiation, similar to the empty vector treated culture (Fig. 5b). We have observed equally well pronounced inhibition of megakaryocytic differentiation effect of DLGAP1 downregulation in UT7/TPO cells (data not shown).

### CDK1 activity ameliorates the centrosomal association of DLGAP1 and attenuates polyploidization processes driven by DLGAP1

**Fig. 6:**
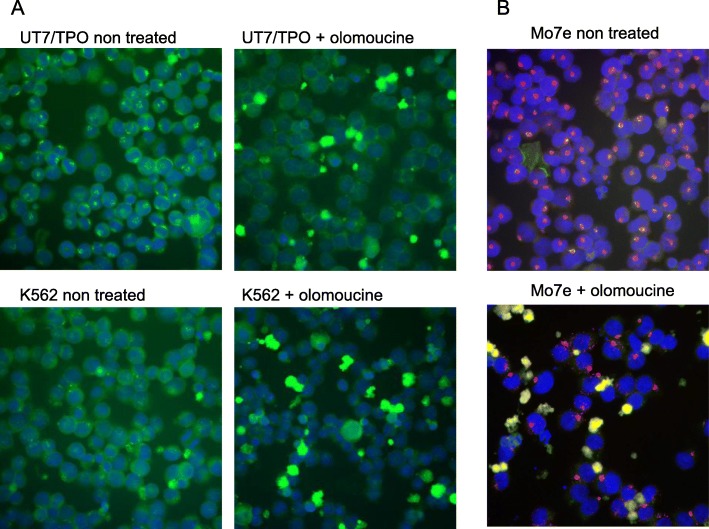
Effects of CDK1 inhibition on DLGAP1 in hematopoietic cells. **a** Immunofluorescent microscopy of UT7/TPO and K562 cells untreated and treated with olomoucine. Native DLGAP1 was detected with specific antibody and stained green, DNA was stained blue with DAPI. **b** Immunofluorescent microscopy of Mo7e cells untreated and treated with olomoucine. Native DLGAP1 and PCM1 were detected with respective specific antibodies and stained green and red respectively. DNA was stained blue with DAPI

A recent finding of Dlgap1 phosphorylation by CDK5 in neural cells leading to its proteasomal degradation^29^ prompted us to test for possible function of CDK1 on DLGAP1 in hematopoietic cells. CDK1 is a functional counterpart of CDK5 in hematopoietic cells where it is the dominant cyclin dependent kinase. The two kinases have the most homologous phosphorylation domains among CDKs resulting in phosphorylation of the same consensus receptor sites. There are multiple consensus CDK1 phosphorylation sites in DLGAP1 protein (Additional file 2). We have tested the effect of CDK1 kinase activity inhibition by olomoucine on DLGAP1 in hematopoietic cell lines. A 3-day treatment of the UT7/TPO and K562 cell cultures with olomoucine resulted in a complete dissociation of DLGAP1 from centrosomes in each case (Fig. 6a). Interestingly, the treatment of cells with olomoucine, did not affect the centrosomal localization of PCM1, as indicated in Mo7e cells (Fig. 6b).

**Fig. 7:**
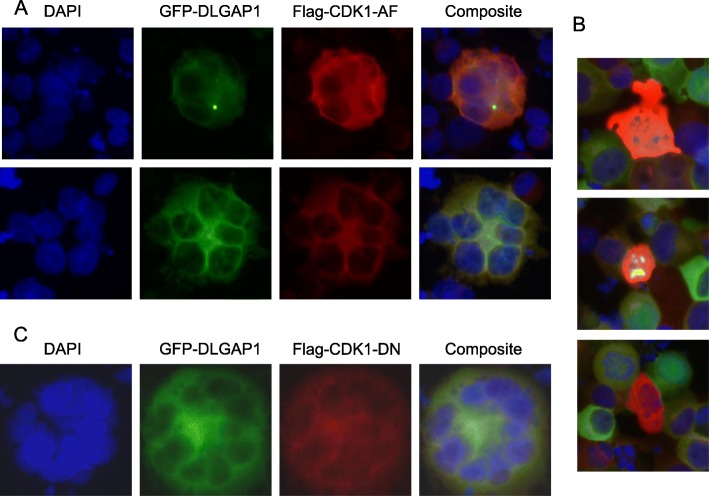
Effects of CDK1 overexpression and downregulation on megakaryocytic polyploidization in cells overexpressing DLGAP1. **a** Immunofluorescent microscopy of UT7/TPO cells overexpressing GFP-DLGAP1 and transfected with constitutively active clone of CDK1 (Flag-CDK1-AF). GFP-DLGAP1 was detected green, the constitutively active CDK1 clone was stained red with anti-Flag antibody and DNA was stained blue with DAPI. **b** Immunofluorescent microscopy of K562 cells overexpressing GFP-DLGAP1 and constitutively active clone of CDK1(Flag-CDK1-AF). The GFP-DLGAP1 was captured using green fluorescence, the constitutively active CDK1 clone was stained red with anti-Flag antibody) and DNA was stained blue with DAPI. **c** Immunofluorescent microscopy of K562 cells overexpressing GFP-DLGAP1 and dominant negative clone of CDK1(Flag-CDK1-ND). The GFP-DLGAP1 was captured using green fluorescence, the dominant negative CDK1 clone was stained red with anti-Flag antibodyand DNA was stained blue with DAPI

We have also tested the effects of constitutively active and dominant negative clones of CDK1 (Flag-CDK1-AF and Flag-CDK1-ND respectively), in hematopoietic cells overexpressing DLGAP1. While both forms of CDK1 colocalized with cytoplasmic DLGAP1, they have opposite effects on the centrosomal accumulation of DLGAP1. The constitutively active construct of CDK1 in UT7/TPO cells supported DLGAP1 localization in centrosomes even in middle stages of megakaryocytic proliferation (Fig. 7a, upper panel). This was not observed under regular growth conditions or otherwise induced polyploidization. Importantly, the centrosomal DLGAP1 was still detectable in the infrequent cases of more advanced ploidy stages observed in the presence of constitutively active CDK1 (Fig. 7a, bottom panel). Importantly, in parallel experiments in K562 cells overexpressing DLGAP1, the constitutively active clone of CDK1 resulted in aberrant polyploidization and defective cells resembling apoptotic events (Fig. 7b).

When the dominant negative form of CDK1 was overexpressed in K562 cells double transfected with the GFP-DLGAP1, no GFP-DLGAP1 concentration was observed in centrosomal areas. Double transfected cells frequently developed very advanced ploidy states in these cultures (Fig. 7c).

## Discussion

Our retroviral- based insertional mutagenesis screen for factors that cooperate with the MPL signaling identified the *DLGAP1* gene as the most prominent candidate. This protein is a member of the Discs-large/Scribble/Lethal Giant Larvae pathway. This pathway controlling of cell proliferation and polarity has been implicated in cancer development [27, 28, 30].

According to the National Center for Biotechnology Information (NCBI) database, the human *DLGAP1* gene contains 25 distinct gt-ag introns and transcription produces 16 different, tissue dependent mRNAs. In one study four alternative *DLGAP1* promoters were identified in mouse hippocampus which coincided with 4 major DLGAP1 isoforms [31].

Among our DLGAP1 RIS candidate clones we suspect that the retroviral insertions upregulate a truncated isoform at the 4th intron, the RISs location. A natural isoform of DLGAP1 exists that corresponds to this expected, RISs generated truncated form. We could also not exclude the possible upregulation of the full length DLGAP1, since mRNA splicing would remove intron 4 along with the integrated provirus. The truncated isoform of DLGAP1 retains the C terminus of full length DLGAP1, which serves as a binding sequence to the PDZ domain of SHANK proteins [32] but lacks all five of the14 amino-acid repeats, conserved in DLGAP family located closer to the amino end of the molecule, and serve to bind the GK domain of DLG proteins [33]. Interestingly, recent studies revealed that SHANK proteins are binding partners of active Rap1 and their silencing triggered increased plasma membrane Rap1 activity, cell spreading, migration and invasion [34]. These SHANK3 driven processes could contribute to cancer progression and metastasis, but they could also support proper seeding and maturation and release of hematopoietic stem cells from the bone marrow niche. Importantly, a sustained activation of Rap1 in mouse hematopoietic stem cells was found to cause the expansion of hematopoietic progenitors, followed by a myeloproliferative disorder [35]. Rap1 is also a critical regulator of platelet adhesiveness and activation and was implicated in megakaryocyte differentiation [36] and platelet activation [37]. Based on our observations of increased DLGAP1 concentration close to cell membrane, we suspect that DLGAP1 binds SHANK and likely affects SHANK silencing of Rap1.

The centrosomal DLGAP1 localization is equally important as centrosomal aberrations, which are a common feature in most solid tumors, are frequently observed in MPN [38, 39], and in myeloid leukemia [40]. Maneville et al., reported DLGAP1 association with PDZ-domain-containing protein DLG1 and with the small G protein Cdc42, where these interactions played fundamental role in centrosome positioning and cell polarization [41]. Both of these processes are critical in cell function as precise positioning of the centrosome is essential during cell division [42], and the orientation of the nucleus–centrosome axis indicates the direction of migration in several cell types [43]. In that respect megakaryocyte migration from the proliferative osteoblastic niche within the bone marrow environment to the capillary-rich vascular niche is an essential step for platelet production. Cdc42 has been shown to be the main player controlling proper megakaryopoiesis [44], and the production of functional platelets [45, 46]. Mouse model studies have found that hematopoietic stem cell aging is associated with elevated activity of Cdc42 in HSCs, resulting in the loss of polarity, among other functional deficits of aged HSCs [47]. HSC aging is correlated with an increased incidence of myeloid malignancy.

DLGAP1 was also implicated in Alzheimer’s and other neurological disorders, where as a direct substrate of CDK5, it undergoes phosphorylation and proteasomal degradation leading to synaptic actin remodeling and ultimately to a synapse loss [29]. Analogous processes may explain our findings of CDK1 and DLGAP1 interaction effects on megakaryocytic polyploidization. The CDK1-DLGAP1 interaction may also be involved in platelet production and activation, where both processes require extensive membrane remodeling.

A recent evidence of a direct DLGAP1 involvement in oncogenic processes comes from studies by Li at al [48], where DLGAP1 was established as a modifier of invasive cancer growth driven by NMDAR signaling. DLGAP1 impact on FMRP protein expression and on HSF1 factor activation led to increased invasiveness of PanNET and pancreatic ductal adenocarcinoma cancer cells [48]. Both FMRP and HSF1 were previously indicated to be upregulated in other cancers [48] and the HSF1 activity in MPN was also recently reported [49]. It is significant that a unique FMRP form was reported to be abundant in human platelets [50].

## Conclusions

These studies and our findings of cell cycle dependent centrosomal manifestation of DLGAP1, its activity in megakaryocytic differentiation, and its sensitivity to and cooperation with hematopoietic relevant kinases point to DLGAP1 involvement in megakaryocyte biology and platelet function. Future experiments would be required to test our speculation that the full length DLGAP1 may play substantial role in early megakaryocyte development to retard proliferation and support polyploidization processes whereas truncated isoforms or other aberrant DLGAP1 forms may give proliferative advantage to hematopoietic cells undergoing neoplastic transformation.

## Additional files

Additional file 1: Selection of MPL dependent K562 cells. (**A**) Line graph of 4 days treatment of plain and MPL driven K562 cells with AG490. (**B**) Bright field microscopy of Wright’s stained of the MGIFMNO transduced K562 cells sorted for GFP. Cells after sorting and before treatment with Imatinib and AG20187. (**C**) Cells from (B) selected for 7 days on Imatinib and AG20187. (PPTX 2449 kb)

Additional file 2: The Retroviral insertion sites localization on human chromosomes and listing of surrounding genes and genomic features. (DOCX 37 kb)

Additional file 3: A sample of expression and copy number data on DLGAP1 in hematologic malignancies from Oncomine Platform (www.oncomine.org). (**A-D**) Expression data. (**E-F**) Copy number data. (PPTX 93 kb)

Additional file 4: (**A**) Motif consensus sequences and localization of phosphorylation sites in human DLGAP1 protein for hematopoietic relevant Tyrosine kinases. (**B**) Motif consensus sequences and localization of phosphorylation sites in human DLGAP1 protein for selected hematopoietic relevant Serine kinases. (DOCX 20 kb)

Additional file 5 Native DLGAP1 in UT7/TPO cells under treatment with hematopoietic relevant Tyrosine kinases inhibitors. (**A**) untreated (+DMSO). (**B**) treated with tyrosine kinase inhibitors AG490, SU6656 and UO126. DLGAP1 was stained green with specific antibody. (**c**) Staining of PCM1 with specific antibody in red and cellular DNA stained blue with DAP!. (PPTX 1546 kb)

Additional file 6: Fluorescent microscopy of cells treated with hematopoietic relevant Tyrosine kinase inhibitors. Native DLGAP1 and PCM1 were labeled with specific antibodies and stained green and red respectively. Cellular DNA was stained blue with DAPI. (PPTX 2791 kb)

## Data Availability

Materials are available upon request.

## Abbreviations

AML: Acute Myeloid Leukemia
BLAST: Basic Local Alignment Search Tool
EGFP: Enhanced Green Fluorescent Protein
ET: Essential Thrombocytosis
IRES: Internal Ribosomal Entry Site
MPN: Myeloproliferative Neoplasms
MSCV: Murine Stem Cell Virus
NCBI: National Center for Biotechnology Information
PMF: Primary Myelofibrosis
PV: Polycythemia Vera
