# Supplementary figures and images for: DLGAP1 directs megakaryocytic growth and differentiation in an MPL dependent manner in hematopoietic cells
Source: Biomark Res. 2019 Jul 8;7:13. doi: 10.1186/s40364-019-0165-z (PMC6615210; doi:10.1186/s40364-019-0165-z)

## Slide 1
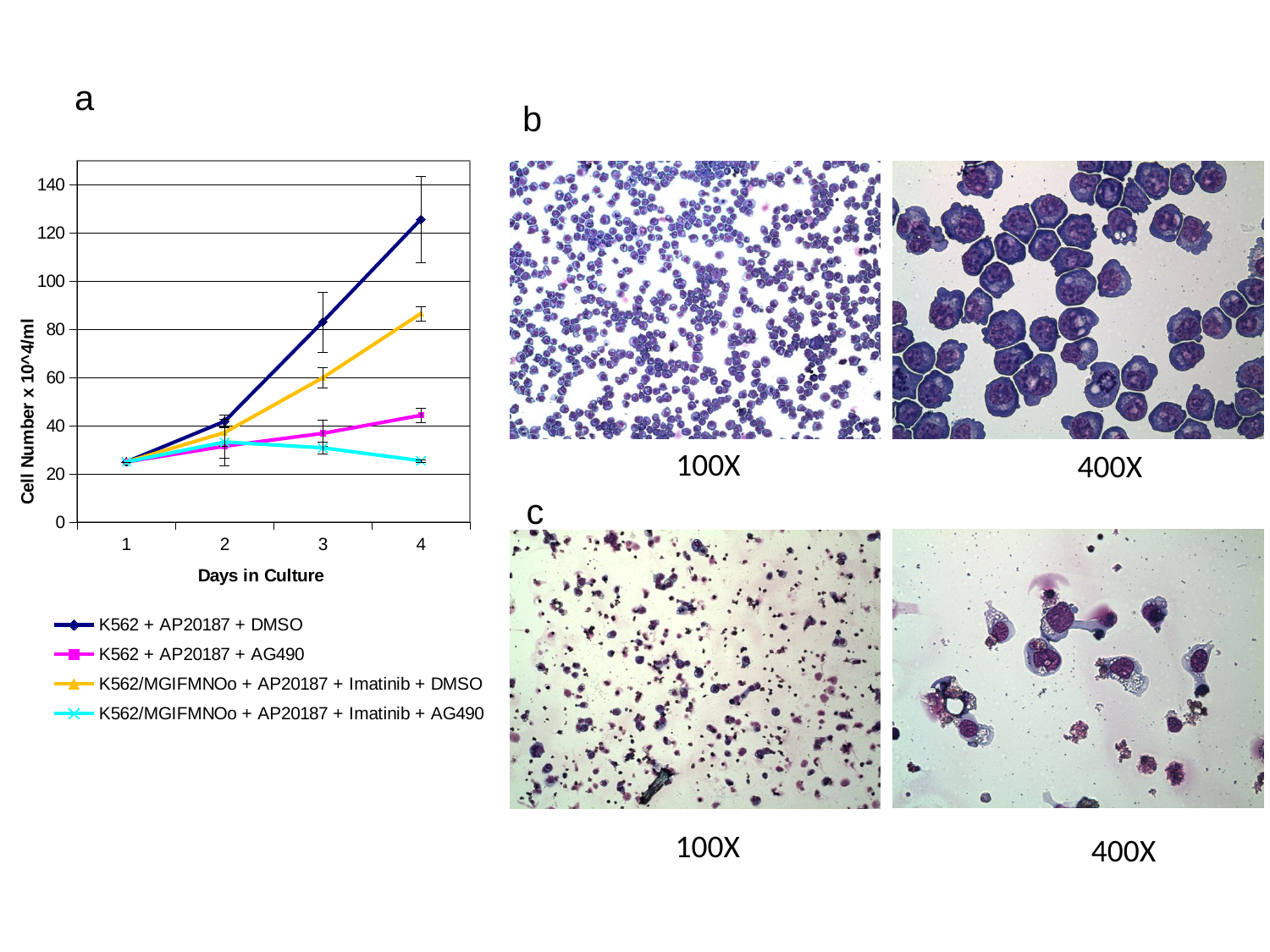

a
b
### Chart
| Category | | | | |
|---|---|---|---|---|
| 1 | 25.0 | 25.0 | 25.0 | 25.0 |
| 2 | 41.833333333333336 | 31.5 | 37.166666666666664 | 33.25 |
| 3 | 83.08333333333333 | 36.833333333333336 | 59.916666666666664 | 30.833333333333332 |
| 4 | 125.66666666666667 | 44.333333333333336 | 86.58333333333333 | 25.5 |
100X
400X
c
100X
400X

Supplement: Supplementary file 1 — Selection of MPL dependent K562 cells. (A) Line graph of 4 days treatment of plain and MPL driven K562 cells with AG490. (B) Bright field microscopy of Wright’s stained of the MGIFMNO transduced K562 cells sorted for GFP. Cells after sorting and before treatment with Imatinib and AG20187. (C) Cells from (B) selected for 7 days on Imatinib and AG20187. (PPTX 2449 kb) [file 40364_2019_165_MOESM1_ESM.pptx]

## Slide 1
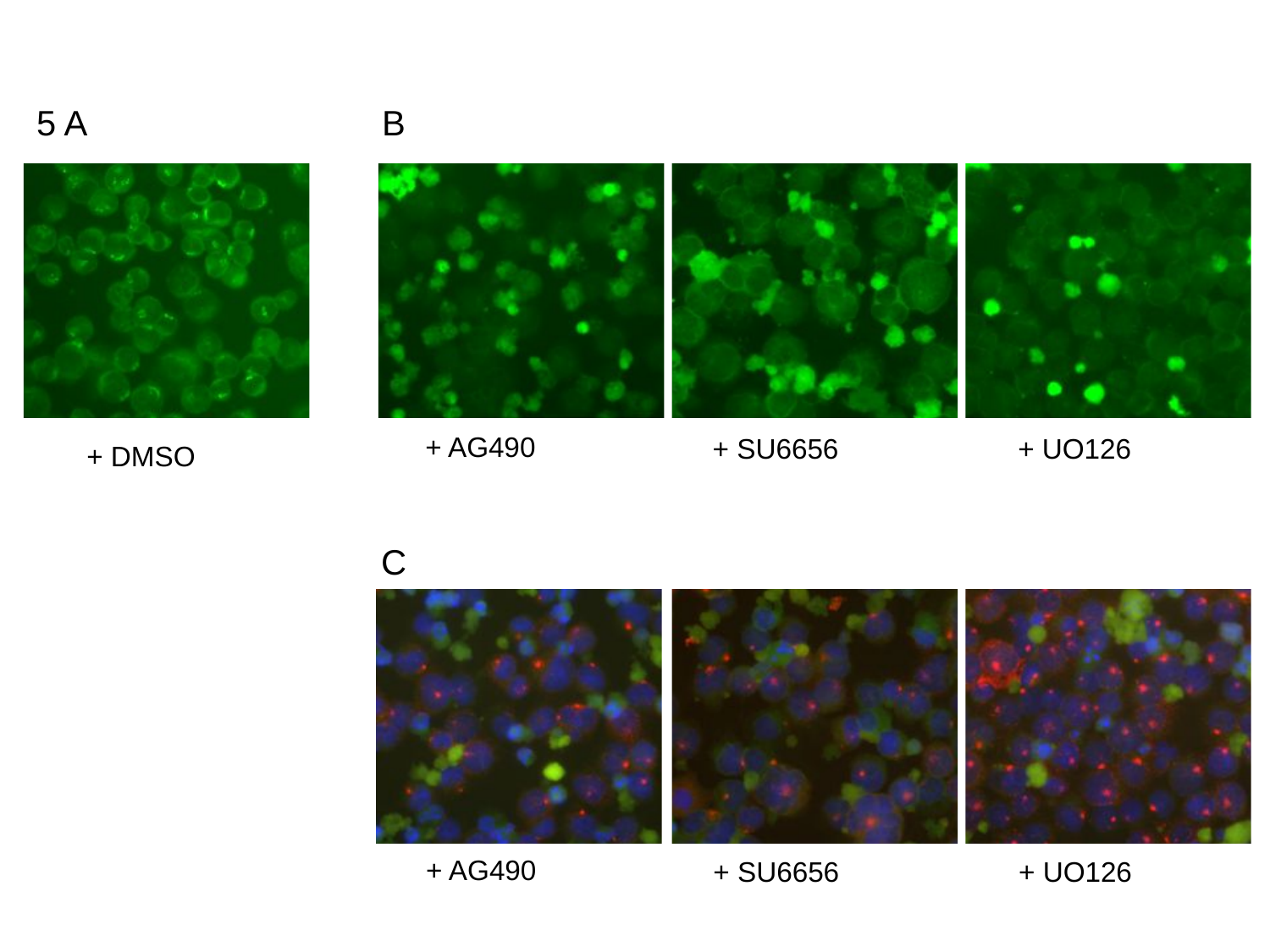

5 A
B
+ AG490
+ SU6656
+ UO126
+ DMSO
C
+ AG490
+ SU6656
+ UO126

Supplement: Supplementary file 5 — Native DLGAP1 in UT7/TPO cells under treatment with hematopoietic relevant Tyrosine kinases inhibitors. (A) untreated (+DMSO). (B) treated with tyrosine kinase inhibitors AG490, SU6656 and UO126. DLGAP1 was stained green with specific antibody. (c) Staining of PCM1 with specific antibody in red and cellular DNA stained blue with DAP!. (PPTX 1546 kb) [file 40364_2019_165_MOESM5_ESM.pptx]

## Slide 1
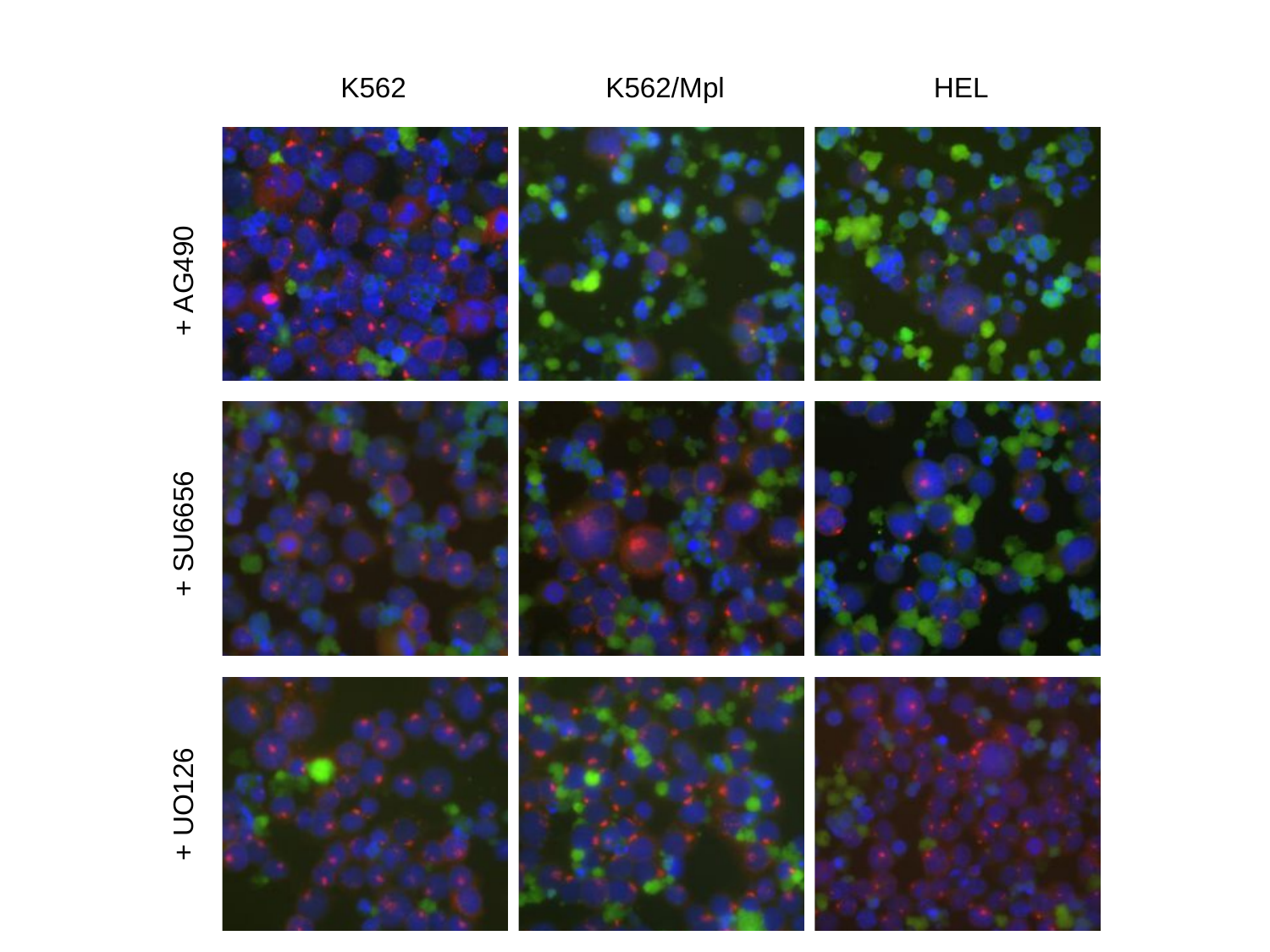

K562
K562/Mpl
HEL
+ AG490
+ SU6656
+ UO126

Supplement: Supplementary file 6 — Fluorescent microscopy of cells treated with hematopoietic relevant Tyrosine kinase inhibitors. Native DLGAP1 and PCM1 were labeled with specific antibodies and stained green and red respectively. Cellular DNA was stained blue with DAPI. (PPTX 2791 kb) [file 40364_2019_165_MOESM6_ESM.pptx]
