# Supplementary material for: DLGAP1 directs megakaryocytic growth and differentiation in an MPL dependent manner in hematopoietic cells
Source: Biomark Res. 2019 Jul 8;7:13. doi: 10.1186/s40364-019-0165-z (PMC6615210; doi:10.1186/s40364-019-0165-z)
Supplement: Supplementary file 2 — The Retroviral insertion sites localization on human chromosomes and listing of surrounding genes and genomic features. (DOCX 37 kb) [file 40364_2019_165_MOESM2_ESM.docx]

| **Trans-duction No** | **DNA Sample Origin** | **RIS Genome Localization** | **Surrounding genes** |
| --- | --- | --- | --- |
| 1 | K562/MGIFMNOo | 1p36.22 | 10293 bp at 5' side: chromosome 1 open reading frame 187 5985 bp at 3' side: angiotensin II receptor-associated protein isoform e |
|  | K562/MGIFMNOo | Xp22.1 | Features in this part of subject sequence: hypothetical protein LOC254158 (CXorf58) |
|  | K562/MGIFMNOo | 12p13 | proacrosin binding protein sp32 precursor aka. OY-TES-1 |
|  | K562/MGIFMNOo | 3q23 | [114135 bp at 5' side: phosphoinositide-3-kinase, catalytic, beta polypeptide  71847 bp at 3' side: forkhead box L2](http://www.ncbi.nlm.nih.gov/entrez/viewer.fcgi?val=88966845&db=Nucleotide&from=44869377&to=44973331&view=gbwithparts&RID=6ADT7S6X014) |
|  | K562/MGIFMNOo | 1q21 | 7563 bp at 5' side: hypothetical protein  2099 bp at 3' side: hypothetical protein (similar to Thromboxane A2 receptor) |
|  | K562/MGIFMNOo | 16p13.13 | RSL1D1 (PBK1 - nucleostemin interacting protein) |
|  | K562/MGIFMNOo | 5q31 | 326053 bp at 5' side: follistatin-like 4  26433 bp at 3' side: hypothetical protein LOC56951 |
|  | K562/MGIFMNOo | 1p36.1 | 23150 bp at 5' side: heparan sulfate proteoglycan 2  57246 bp at 3' side: elastase 3B preproprotein preproprotein |
|  | K562/MGIFMNOo | 9q22 | 44083 bp at 5' side: hemogen  873 bp at 3' side: acidic (leucine-rich) nuclear phosphoprotein 32 family, m… |
|  | K562/MGIFMNOo | 7p14.3 | 12231 bp at 5' side: kelch repeat and BTB (POZ) domain containing 2  65290 bp at 3' side: FK506 binding protein 9 |
|  | K562/MGIFMNOo | 18p11.2 | 5706 bp at 5' side: twisted gastrulation  107696 bp at 3' side: ralA binding protein 1 |
|  | K562/MGIFMNOo | 9q31 | [nipsnap homolog 3A](http://www.ncbi.nlm.nih.gov/entrez/viewer.fcgi?val=89030004&db=Nucleotide&from=14831279&to=14842824&view=gbwithparts&RID=6AMHDEXE01R) |
|  | K562/MGIFMNOo | 11q13 | 126450 bp at 5' side: keratin associated protein 5-11  77760 bp at 3' side: hypothetical protein LOC55199 isoform 3 |
|  | K562/MGIFMNOo | 7q22 | 10490 bp at 5' side: ephrin receptor EphB4 precursor  16034 bp at 3' side: solute carrier family 12 (potassium/chloride transporters… |
|  | K562/MGIFMNOo | 1p34 | 738325 bp at 5' side: POU domain, class 3, transcription factor 1  54013 bp at 3' side: Ras-related GTP binding C |
|  | K562/MGIFMNOo | 11p15 | epsilon globin |
|  | K562/MGIFMNOo | 11p15 | 23 bp at 5' side: A-gamma globin  3027 bp at 3' side: G-gamma globin |
|  | K562/MGIFMNOo | 14q24 | 55240 bp at 5' side: hypothetical protein  29512 bp at 3' side: hypothetical protein LOC9766 |
|  | K562/MGIFMNOo | 19q13.2 | 15630 bp at 5' side: spectrin, beta, non-erythrocytic 4 isoform sigma5  461 bp at 3' side: SH3KBP1 binding protein 1(CBL interacting protein) |
|  | K562/MGIFMNOo | 10q22 | cadherin related 23 isoform 1 precursor  cadherin related 23 isoform 2 precursor |
|  | K562/MGIFMNOo | 22q13 | 29169 bp at 5' side: tubulin tyrosine ligase-like family, member 1 isoform a 18987 bp at 3' side: BCL2-interacting killer |
|  | K562/MGIFMNOo | Multiple | unplaced contig ref\|NT_113961.1 matching several chromosome ends |
|  | K562/MGIFMNOo | 5p15.2 | membrane-associated ring finger (C3HC4) 6 |
|  | K562/MGIFMNOo | 7p13 | 31608 bp at 5' side: cell cycle progression 2 protein isoform 2 aka transforming growth factor beta regulator 4 (TBRG4) 16513 bp at 3' side: receptor activity modifying protein 3 precursor |
|  |  |  |  |
| 2 | UT7-TPO/MGIFMNOo | 11q24 | Cbl-interacting protein Sts-1 |
|  | UT7-TPO/MGIFMNOo | 6p22.1 | 70761 bp at 5' side: zinc finger protein 192  2067 bp at 3' side: zinc finger protein 193 |
|  | UT7-TPO/MGIFMNOo | 21q11.2 | 117830 bp at 5' side: similar to hCG1818297  203240 bp at 3' side: nuclear receptor interacting protein 1 |
|  | UT7-TPO/MGIFMNOo | 17q25 | 7772 bp at 5' side: hypothetical protein  9023 bp at 3' side: hypothetical protein |
|  | UT7-TPO/MGIFMNOo | 17q12 | 343 bp at 5' side: schlafen family member 14  7912 bp at 3' side: similar to mCG146274 |
|  | UT7-TPO/MGIFMNOo | 7q33 | transcriptional intermediary factor 1 alpha isoform b  transcriptional intermediary factor 1 alpha isoform a |
|  | UT7-TPO/MGIFMNOo | 7p13 | 31562 bp at 5' side: cell cycle progression 2 protein isoform 2  16470 bp at 3' side: receptor activity modifying protein 3 precursor |
|  | UT7-TPO/MGIFMNOo | 7p14.2 | 141008 bp at 5' side: dpy-19-like 1  22976 bp at 3' side: T-box transcription factor TBX20 isoform A |
|  | UT7-TPO/MGIFMNOo | 10q22 | 59471 bp at 5' side: hypothetical protein  789014 bp at 3' side: hypothetical protein |
|  | UT7-TPO/MGIFMNOo | 15q15 | Bcl2 modifying factor isoform bmf-1  Bcl2 modifying factor isoform bmf-2 |
|  | UT7-TPO/MGIFMNOo | 22q13 | 29059 bp at 5' side: tubulin tyrosine ligase-like family, member 1 isoform a  18814 bp at 3' side: BCL2-interacting killer |
|  |  |  |  |
| 3 | UT7/TPO/MGIFMNOo | 1q21 | 16757 bp at 5' side: S100 calcium binding protein A7-like 1  415 bp at 3' side: S100 calcium binding protein A7-like 2 |
|  | UT7/TPO/MGIFMNOo | 6q26 | 34227 bp at 5' side: 1-acylglycerol-3-phosphate O-acyltransferase 4  83017 bp at 3' side: parkin isoform 1 |
|  | UT7/TPO/MGIFMNOo | 14q32 | 139060 bp at 5' side: hypothetical protein  47101 bp at 3' side: delta-like 1 homolog |
|  | UT7/TPO/MGIFMNOo | 12q13 | 6749 bp at 5' side: nuclear factor (erythroid-derived 2), 45kDa (NFEW2)  22976 bp at 3' side: coatomer protein complex, subunit zeta 1(COPZ1) containing miR148B |
|  | UT7/TPO/MGIFMNOo | 18p11.3 | discs large homolog-associated protein 1 isoform alpha  discs large homolog-associated protein 1 isoform beta |
|  | UT7/TPO/MGIFMNOo | 20q11.2 | 205393 bp at 5' side: hypothetical protein LOC140688  22572 bp at 3' side: COMM domain containing 7 isoform 2 |
|  |  |  |  |
| 4 | K562/MFhuMIGNOo | 21q22 | MORC family CW-type zinc finger 3 |
|  | K562/MFhuMIGNOo | 14q24 | 2421 bp at 5' side: butyrate response factor 1  156 bp at 3' side: hypothetical protein LOC400223 |
|  | K562/MFhuMIGNOo | 18p11.3 | discs large homolog-associated protein 1 isoform alpha  discs large homolog-associated protein 1 isoform beta |
|  | K562/MFhuMIGNOo | 20q11.2 | 205756 bp at 5' side: hypothetical protein LOC140688  22572 bp at 3' side: COMM domain containing 7 isoform 2 82kb on 3' side DNMT3B |
|  | K562/MFhuMIGNOo | 19q13.1 | PKC-potentiated PP1 inhibitory protein (PPP1R14A) (smooth muscle myosin phosphatase inhibitory protein) |
|  | K562/MFhuMIGNOo | 7q33 | transcriptional intermediary factor 1 alpha isoform b  transcriptional intermediary factor 1 alpha isoform a |
|  | K562/MFhuMIGNOo | 1q23 | 14831 bp at 5' side: heat shock 70kDa protein 6 (HSP70B')  1312 bp at 3' side: Fc fragment of IgG, low affinity IIIa, receptor for (CD16) |
|  | K562/MFhuMIGNOo | 14q23.1 | 13849 bp at 5' side: translocase of inner mitochondrial membrane 9 homolog  2126 bp at 3' side: talpid3 protein |
|  |  |  |  |
| 5 | UT7/MFhuMIGNOo | 11p11.2 | CD82 antigen isoform 2 |
|  | UT7/MFhuMIGNOo | 14q13 | hypothetical protein LOC283635 isoform 1 upregulation of hypothetical protein LOC283635 isoform 2 |
|  | UT7/MFhuMIGNOo | 3q21 | 41867 bp at 5' side: putative GR6 protein  4281 bp at 3' side: ribophorin I precursor |
|  | UT7/MFhuMIGNOo | Yq12/Xq28 | interleukin 9 receptor isoform 1 precursor  interleukin 9 receptor isoform 2 |
|  | UT7/MFhuMIGNOo | 6p21.3 | 3776 bp at 5' side: G6f protein  500 bp at 3' side: lymphocyte antigen 6 complex G6D |
|  |  |  |  |
| 6 | UT7/MFhuMIGNOo | 10q23 | 34836 bp at 5' side: alpha 2 actin  6689 bp at 3' side: tumor necrosis factor receptor superfamily, member 6 isof |
|  | UT7/MFhuMIGNOo | 15q15 | 697 bp at 5' side: erythrocyte membrane protein band 4.2  11229 bp at 3' side: transglutaminase 5 isoform 2 |
|  | UT7/MFhuMIGNOo | 3q21 | 25177 bp at 5' side: protein tyrosine phosphatase-like (proline instead of cat...  3330 bp at 3' side: kinase related protein, telokin isoform 8 |
|  | UT7/MFhuMIGNOo | 11p11.2 | CD82 antigen isoform 2 |
|  | UT7/MFhuMIGNOo | 14q13 | hypothetical protein LOC283635 isoform 1  hypothetical protein LOC283635 isoform 2 (C14ofr24) |
|  | UT7/MFhuMIGNOo | 4q31.3 | 107384 bp at 5' side: GAJ protein  34631 bp at 3' side: hypothetical protein LOC23240 |
|  |  |  |  |
| 7 | K562/MGIFMNOo | 9q34 | ubiquitin associated domain containing 1 (UBAC1) |
|  | K562/MGIFMNOo | 4p14 | 433 bp at 5' side: hypothetical protein  15447 bp at 3' side: Kruppel-like factor 3 (basic) |
|  | K562/MGIFMNOo | 20q13.1 | zinc finger, MYND-type containing 8 isoform b  zinc finger, MYND-type containing 8 isoform a |
|  | K562/MGIFMNOo | 15q15 | 16765 bp at 5' side: tumor protein p53 binding protein 1 (TP53BP1)  11290 bp at 3' side: microtubule-associated protein 1A |
|  | K562/MGIFMNOo | 10p13 | chromosome 10 open reading frame 97 |
|  | K562/MGIFMNOo | 15q21.3 | hypothetical protein |
|  |  |  |  |
| 8 | UT7/MGIFMNOo | 6p11 | 505023 bp at 5' side: hypothetical protein |
|  | UT7/MGIFMNOo | 17p13 | 2267 bp at 5' side: ribosomal protein L26 (RPL26)  8140 bp at 3' side: similar to rCG35065 |
|  | UT7/MGIFMNOo | 12p13 | 4906 bp at 5' side: cyclin-dependent kinase inhibitor 1B  60972 bp at 3' side: apolipoprotein L domain containing 1 |
|  | UT7/MGIFMNOo | 10q26 | 27381 bp at 5' side: PDZ domain containing 8  139792 bp at 3' side: empty spiracles homeobox 2 |
|  | UT7/MGIFMNOo | 9p22 | 56822 bp at 5' side: FRAS1 related extracellular matrix 1  245365 bp at 3' side: hypothetical protein LOC158219 |
|  | UT7/MGIFMNOo | 10q11.2 | 12307 bp at 5' side: beta-microseminoprotein isoform b precursor  4373 bp at 3' side: nuclear receptor coactivator 4 |
|  | UT7/MGIFMNOo | 1q22 | lamin A/C isoform 1 precursor  lamin A/C isoform 3 |
|  | UT7/MGIFMNOo | 11q12 | 14073 bp at 5' side: membrane-spanning 4-domains, subfamily A, member 2  62381 bp at 3' side: membrane-spanning 4-domains, subfamily A, member 6A isofo… |
|  |  |  |  |
| 9 |  | 4p14 | 458 bp at 5' side: hypothetical protein  15447 bp at 3' side: Kruppel-like factor 3 (basic) |
|  | K562/MGIFMNOo | 20q11.2 | 205811 bp at 5' side: hypothetical protein LOC140688  22572 bp at 3' side: COMM domain containing 7 isoform 2 |
|  | K562/MGIFMNOo | 17p13 | 1308 bp at 5' side: ribosomal protein L26  8140 bp at 3' side: similar to rCG35065 |
|  | K562/MGIFMNOo | 18p11.3 | discs large homolog-associated protein 1 isoform alpha  discs large homolog-associated protein 1 isoform beta |
|  | K562/MGIFMNOo | 15q15 | 16765 bp at 5' side: tumor protein p53 binding protein 1  11497 bp at 3' side: microtubule-associated protein 1A |
|  | K562/MGIFMNOo | 1p36 | 10293 bp at 5' side: chromosome 1 open reading frame 187  5728 bp at 3' side: angiotensin II receptor-associated protein isoform e |
|  |  |  |  |
| 10 | K562/MGIFMNOo | 4p14 | 458 bp at 5' side: hypothetical protein  15447 bp at 3' side: Kruppel-like factor 3 (basic) |
|  | K562/MGIFMNOo | 20q11.2 | 205811 bp at 5' side: hypothetical protein LOC140688  22572 bp at 3' side: COMM domain containing 7 isoform 2 |
|  | K562/MGIFMNOo | 17p13 | 1308 bp at 5' side: ribosomal protein L26  8140 bp at 3' side: similar to rCG35065 |
|  | K562/MGIFMNOo | 18p11.3 | discs large homolog-associated protein 1 isoform alpha  discs large homolog-associated protein 1 isoform beta |
|  |  |  |  |
| 11 | K562/MFhuMIGNOo | 3q21 | 41873 bp at 5' side: putative GR6 protein  4281 bp at 3' side: ribophorin I precursor |
|  | K562/MFhuMIGNOo | 18p11.3 | discs large homolog-associated protein 1 isoform alpha  discs large homolog-associated protein 1 isoform beta |
|  | K562/MFhuMIGNOo | 20q11.2 | 205547 bp at 5' side: hypothetical protein LOC140688  22572 bp at 3' side: COMM domain containing 7 isoform 2 |
|  | K562/MFhuMIGNOo | 15q15 | 16765 bp at 5' side: tumor protein p53 binding protein 1  11447 bp at 3' side: microtubule-associated protein 1A |
|  | K562/MFhuMIGNOo | 17p13 | 1308 bp at 5' side: ribosomal protein L26  8140 bp at 3' side: similar to rCG35065 |
|  | K562/MFhuMIGNOo | 4p14 | 458 bp at 5' side: hypothetical protein  15447 bp at 3' side: Kruppel-like factor 3 (basic) |
|  |  |  |  |
| 12 | K562/MGIFMNOo | 1p36 | 10293 bp at 5' side: chromosome 1 open reading frame 187  5728 bp at 3' side: angiotensin II receptor-associated protein isoform e |
|  | K562/MGIFMNOo | 4p14 | 458 bp at 5' side: hypothetical protein  15447 bp at 3' side: Kruppel-like factor 3 (basic) |
|  | K562/MGIFMNOo | 15q15 | 16765 bp at 5' side: tumor protein p53 binding protein 1  11447 bp at 3' side: microtubule-associated protein 1A |
|  | K562/MGIFMNOo | 17p13 | 1308 bp at 5' side: ribosomal protein L26  8140 bp at 3' side: similar to rCG35065 |
|  | K562/MGIFMNOo | 20q11.2 | 205547 bp at 5' side: hypothetical protein LOC140688  22572 bp at 3' side: COMM domain containing 7 isoform 2 |
|  | K562/MGIFMNOo | 10q26 | 27381 bp at 5' side: PDZ domain containing 8  139792 bp at 3' side: empty spiracles homeobox 2 |
|  |  |  |  |
| 13 | UT7/MGIFMNOo | 17p13 | 1308 bp at 5' side: ribosomal protein L26  8140 bp at 3' side: similar to rCG35065 |
|  | UT7/MGIFMNOo | 20q11.2 | 205547 bp at 5' side: hypothetical protein LOC140688  22572 bp at 3' side: COMM domain containing 7 isoform 2 |
|  | UT7/MGIFMNOo | 9q34 | ubiquitin associated domain containing 1 (UBAC1) |
|  | UT7/MGIFMNOo | 18p11.3 | discs large homolog-associated protein 1 isoform alpha  discs large homolog-associated protein 1 isoform beta |
|  | UT7/MGIFMNOo | 15q15 | 16765 bp at 5' side: tumor protein p53 binding protein 1  11290 bp at 3' side: microtubule-associated protein 1A |
|  | UT7/MGIFMNOo | 3q21 | 41873 bp at 5' side: putative GR6 protein  4281 bp at 3' side: ribophorin I precursor |
|  |  |  |  |
| 14 | K562/MGIFMNOo | 17p13 | 1308 bp at 5' side: ribosomal protein L26  8140 bp at 3' side: similar to rCG35065 |
|  | K562/MGIFMNOo | 18p11.3 | discs large homolog-associated protein 1 isoform alpha  discs large homolog-associated protein 1 isoform beta |
|  | K562/MGIFMNOo | 20q11.2 | 205547 bp at 5' side: hypothetical protein LOC140688  22572 bp at 3' side: COMM domain containing 7 isoform 2 |
|  | K562/MGIFMNOo | 9q34 | ubiquitin associated domain containing 1 (UBAC1) |
|  | K562/MGIFMNOo | 4p14 | 458 bp at 5' side: hypothetical protein  15447 bp at 3' side: Kruppel-like factor 3 (basic) |
|  | K562/MGIFMNOo | 14q13 | hypothetical protein LOC283635 isoform 1  hypothetical protein LOC283635 isoform 2 (C14ofr24) |
|  | K562/MGIFMNOo | 2q13 | 12835 bp at 5' side: tubulin tyrosine ligase  690 bp at 3' side: RNA polymerase I polypeptide B |
|  | K562/MGIFMNOo | 15q15 | 16765 bp at 5' side: tumor protein p53 binding protein 1  11290 bp at 3' side: microtubule-associated protein 1A |
|  |  |  |  |
| 15 | K562/MGIFMNOo | 19q12 | Disrupts BC068609 |
|  | K562/MGIFMNOo | 2q11.2 | 27908 bp at 5' side: hypothetical protein LOC51252  159156 bp at 3' side: similar to hCG1732629 |
|  | K562/MGIFMNOo | 9q34 | 116816 bp at 5' side: similar to hCG1654759  13528 bp at 3' side: 1A6/DRIM (down-regulated in metastasis) interacting protein |
|  | K562/MGIFMNOo | 18p11.3 | discs large homolog-associated protein 1 isoform alpha  discs large homolog-associated protein 1 isoform beta |
|  |  |  |  |
| 16 | K562/MGIFMNOo | 1q42 | polypeptide N-acetylgalactosaminyltransferase 2 (GALNT2) |
|  | K562/MGIFMNOo | 1q42 | polypeptide N-acetylgalactosaminyltransferase 2 (GALNT2) |
|  | K562/MGIFMNOo | 1q42 | 35348 bp at 5' side: similar to LOC645339 protein  15208 bp at 3' side: similar to hCG1817424 isoform 2 |
|  | K562/MGIFMNOo | 3q23 | 37758 bp at 5' side: progestin and adipoQ receptor family member IX  249 bp at 3' side: U2-associated SR140 protein |
|  | K562/MGIFMNOo | 1p36.2 | 10293 bp at 5' side: chromosome 1 open reading frame 187  5725 bp at 3' side: angiotensin II receptor-associated protein isoform e |
|  |  |  |  |
| 17 | K562/MGIFMNOo | 14q24 | 14347 bp at 5' side: chromosome 14 open reading frame 4  63152 bp at 3' side: KIAA1737 protein |
|  | K562/MGIFMNOo | 14q24 | 14347 bp at 5' side: chromosome 14 open reading frame 4  63152 bp at 3' side: KIAA1737 protein |
|  | K562/MGIFMNOo | 3q29 | 146062 bp at 5' side: discs, large homolog 1 isoform 2  68088 bp at 3' side: 3-hydroxybutyrate dehydrogenase precursor |
|  | K562/MGIFMNOo | 3q29 | 146062 bp at 5' side: discs, large homolog 1 isoform 2  68088 bp at 3' side: 3-hydroxybutyrate dehydrogenase precursor |
|  | K562/MGIFMNOo | 12q22 | CASP2 and RIPK1 domain containing adaptor with death domain |
|  | K562/MGIFMNOo | 12q22 | CASP2 and RIPK1 domain containing adaptor with death domain |
|  |  |  |  |
| 18 | K562/MGIFMNOo | 3q29 | 5397 bp at 5' side: hypothetical protein LOC152002  2790 bp at 3' side: centaurin, beta 2, Disrupts ACAP2 ArfGAP (CENTB2) |
|  | K562/MGIFMNOo | 4p12 | tec protein tyrosine kinase |
|  | K562/MGIFMNOo | Xp11.3 | 3851 bp at 5' side: ubiquitously-expressed transcript isoform 2  38002 bp at 3' side: similar to hCG29146 |
|  | K562/MGIFMNOo | 8q24.1 | 179028 bp at 5' side: myc proto-oncogene protein  1296885 bp at 3' side: hypothetical protein |
|  | K562/MGIFMNOo | 12p13 | TEA domain family member 4 isoform 1  TEA domain family member 4 isoform 2 |
|  | K562/MGIFMNOo | 12q24.1 | ATPase, Ca++ transporting, cardiac muscle, slow twitch 2 ...  ATPase, Ca++ transporting, cardiac muscle, slow twitch 2 |
|  |  |  |  |
| 19 | K562/MGIFMNOo | 20p13 | 14009 bp at 5' side: mitochondrial ribosomal protein S26  9692 bp at 3' side: oxytocin-neurophysin I preproprotein |
|  | K562/MGIFMNOo | 21q21 | ATP synthase, H+ transporting, mitochondrial F0 complex |
|  | K562/MGIFMNOo | 8q24.2 | 120226 bp at 5' side: hypothetical protein LOC51571  54989 bp at 3' side: development and differentiation enhancing factor 1 (ASAP1) |
|  | K562/MGIFMNOo | 5q35 | CCR4-NOT transcription complex, subunit 6 |
|  | K562/MGIFMNOo | 5p13 | 50724 bp at 5' side: cadherin 6, type 2 preproprotein  27323 bp at 3' side: ribonuclease III, nuclear isoform 2 |
|  |  |  |  |
| 20 | K562/MGIFMNOo | 1p34.2 | 9668 bp at 5' side: coiled-coil domain containing 23  Disrupts erythroblast membrane-associated protein (ERMAP) |
|  | K562/MGIFMNOo | 17q21 | integrin beta chain, beta 3 precursor |
|  | K562/MGIFMNOo | 9q34.11 | 27174 bp at 5' side: zyg-11 homolog B (C. elegans)-like  4564 bp at 3' side: TBC1 domain family, member 13 |
|  | K562/MGIFMNOo | 4p16.3 | 11969 bp at 5' side: Wolf-Hirschhorn syndrome candidate 2 protein  20862 bp at 3' side: similar to hCG1642661 |
|  |  |  |  |
| 21 | K562/MGIFMNOo | 11q12 | 31230 bp at 5' side: olfactory receptor, family 4, subfamily D, member 9  29248 bp at 3' side: oxysterol binding protein |
|  | K562/MGIFMNOo | 1q42 | polypeptide N-acetylgalactosaminyltransferase 2 |
|  | K562/MGIFMNOo | 16p13.3 | 11681 bp at 5' side: rhomboid family 1  708 bp at 3' side: N-methylpurine-DNA glycosylase isoform b |
|  | K562/MGIFMNOo | 20q13.1 | protein tyrosine phosphatase, non-receptor type 1 |
|  | K562/MGIFMNOo | 10p15 | phosphofructokinase, platelet |
|  |  |  |  |
| 22 | K562/MGIFMNOo | 1q12 | phosphodiesterase 4D interacting protein isoform 1  phosphodiesterase 4D interacting protein isoform 2 |
|  | K562/MGIFMNOo | 17p13 | tyrosine 3/tryptophan 5 -monooxygenase activation protein (YWHAE) 20kb from 3' end CRK oncongene (negative orientation) |
|  | K562/MGIFMNOo | 19p13.1 | 9730 bp at 5' side: jun D proto-oncogene  15752 bp at 3' side: U6 snRNA-associated Sm-like protein 4 |
|  | K562/MGIFMNOo | 7q22 | 184592 bp at 5' side: myosin light chain 2, precursor lymphocyte-specific  2211 bp at 3' side: cut-like homeobox 1 isoform c |
|  | K562/MGIFMNOo | 1p34.3 | 10847 bp at 5' side: serine/threonine kinase 40  21539 bp at 3' side: LSM10, U7 small nuclear RNA associated |
|  | K562/MGIFMNOo | 6p22 | 266 bp at 5' side: histone cluster 1, H4b  4025 bp at 3' side: histone cluster 1, H3b |
|  |  |  |  |
| 23 | K562/MGIFMNOo | 3p25.3 | 92973 bp at 5' side: lipoma HMGIC fusion partner-like 4  2965 bp at 3' side: myotubularin related protein 14 (MTMR14), transcript variant 2 |
|  | K562/MGIFMNOo | 6p.25.3 | ~9kb at 5' end GDP-mannose 4,6-dehydratase (GMDS) |
|  | K562/MGIFMNOo | 16q22 | 31079 bp at 5' side: ST3 beta-galactoside alpha-2,3-sialyltransferase 2  33553 bp at 3' side: fucokinase |
|  |  |  |  |
| 24 | K562/MGIFMNOo | 11q23.3 | ~20kb at 5'end DEAD (Asp-Glu-Ala-Asp) box polypeptide 6 (DDX6), |
|  | K562/MGIFMNOo | 18p11.3 | discs large homolog-associated protein 1 isoform alpha  discs large homolog-associated protein 1 isoform beta |
|  | K562/MGIFMNOo | 9q22 | Hemogen (EDAG; EDAG-1; HEMGN) |
|  | K562/MGIFMNOo | 10p12 | phosphoribosyl transferase domain containing 1 (PRTFDC1) |
|  |  |  |  |
| 25 | K562/MGIFMNOo | 4q31.21 | OTU domain containing 4 (OTUD4) |
|  | K562/MGIFMNOo | 2q11.2 | LOC51252 |
|  | K562/MGIFMNOo | 15q14 | ~50kb at 5’ end golgi autoantigen, golgin subfamily a, 8B (GOLGA8B) |
|  | K562/MGIFMNOo | 12q12 | YY1 associated factor 2 (YAF2) |
|  | K562/MGIFMNOo | 4p15.2 | stromal interaction molecule 2 |
|  |  |  |  |
| 26 | K562/MGIFMNOo | Multiple | unplaced contig ref\|NT_167221.1 matching several chromosome ends ~7 to 150kb from chomosome ends - in several chromosomes |
|  | K562/MGIFMNOo | 4q31.3 | ~6kb 5’ to LRBA, ~70kb 3’ to RPS3A |
|  | K562/MGIFMNOo | 20q13.13 | ~3.5kb at 5’ end LOC100130589 |
|  | K562/MGIFMNOo | 22q12.1 | ~1kb at 3’end crystallin, beta A4 (CRYBA4) |
|  | K562/MGIFMNOo | 7p22.3 | C7orf20 |
|  | K562/MGIFMNOo | 20q13.1 | protein tyrosine phosphatase, non-receptor type 1 (PTPN1) |
|  | K562/MGIFMNOo | 4p14 | ras homolog gene family, member H (RHOH) |
|  | K562/MGIFMNOo | 11p12 | no close features |
|  | K562/MGIFMNOo | 5p12 | zinc finger protein 131 (ZNF131) |
|  |  |  |  |
| 27 | K562/MGIFMNOo | 9q34 | ABO blood group (alpha 1-3-N-acetylgalactosaminyltransferase |
|  | K562/MGIFMNOo | 16q24 | 45881 bp at 5' side: hypothetical protein XP_002344166  18992 bp at 3' side: chromatin licensing and DNA replication factor 1 (CDT1) |
|  | K562/MGIFMNOo | 5q33 | 50bp at 3'end annexin A6 (ANXA6) |
|  | K562/MGIFMNOo | 5p15.2 | carboxymethylenebutenolidase homolog (CMBL) |
|  |  |  |  |
| 28 | K562/MGIFMNOo | 16q12.1 | 35023 bp at 5' side: PAP associated domain containing 5 isoform b  23689 bp at 3' side: adenylate cyclase 7 |
|  | K562/MGIFMNOo | 1q21.3 | ~500bp at 3'end family with sequence similarity 63, member A (FAM63A) ~1kb at 5" end prune homolog (PRUNE), |
|  | K562/MGIFMNOo | 5q31 | ~4kb at 3’ end cyclin-dependent kinase-like 3 (CDKL3) ~500bp at 5’ end from ubiquitin-conjugating enzyme E2B - RAD6 homolog (UBE2B) 950kb from hsa-mir-1289-2 |
|  | K562/MGIFMNOo | 3q26.32 | no close features |
|  | K562/MGIFMNOo | 3p21 | coiled-coil domain containing 12 (CCDC12) |
|  |  |  |  |
| 29 | K562/MGIFMNOo | Xp11.4 | 40700 bp at 5' side: ubiquitin specific protease 9, X-linked isoform 4  332883 bp at 3' side: mediator complex subunit 14 |
|  | K562/MGIFMNOo | 4q12 | 289049 bp at 5' side: platelet-derived growth factor receptor alpha precursor  72763 bp at 3' side: v-kit Hardy-Zuckerman 4 feline sarcoma viral oncogene hom… |
|  | K562/MGIFMNOo | 7q22.3 | 36154 bp at 5' side: cAMP-dependent protein kinase, regulatory subunit beta 2  104919 bp at 3' side: phosphoinositide-3-kinase, catalytic, gamma polypeptide |
|  | K562/MGIFMNOo | 9q34 | ubiquitin associated domain containing 1 (UBAC1) |
|  | K562/MGIFMNOo | 19p13.3 | 71327 bp at 5' side: olfactory receptor, family 4, subfamily F, member 17  98359 bp at 3' side: phosphatidic acid phosphatase type 2C isoform 1 Disrupts LOC729061 |
|  | K562/MGIFMNOo | 4p16.3 | zinc finger protein 595 |
|  | K562/MGIFMNOo | 3q25 | 239979 bp at 5' side: purinergic receptor P2Y1  135677 bp at 3' side: muscleblind-like 1 isoform d |
|  |  |  |  |
| 30 | K562/MGIFMNOo | 2p11.2 | 29585 bp at 5' side: lysine-rich coiled-coil 1  8721 bp at 3' side: SET and MYND domain containing 1 (SMYD1) |
|  | K562/MGIFMNOo | 11p15 | 1794 bp at 5' side: similar to PRO2987  11652 bp at 3' side: epsilon globin |
|  |  |  |  |
| 31 | K562/MGIFMNOo | 7q31.3 | neuronal cell adhesion molecule isoform B precursor (NRCAM)  neuronal cell adhesion molecule isoform A precursor |
|  | K562/MGIFMNOo | 10q11.2 | sphingomyelin synthase 1 |
|  | K562/MGIFMNOo | 19p13.11 | GATA zinc finger domain containing 2A (GATAD2A) |
|  | K562/MGIFMNOo | 14q11.2 | poly (ADP-ribose) polymerase family, member 2 isoform 1 (PARP2)  poly (ADP-ribose) polymerase family, member 2 isoform 2 10kb at 5’end cyclin B1 interacting protein 1 |
|  | K562/MGIFMNOo | 12q13.13 | 19579 bp at 5' side: nuclear factor (erythroid-derived 2), 45kDa (NFE2)  10074 bp at 3' side: coatomer protein complex, subunit zeta 1 (COPZ1) containing miR148B |
|  |  |  |  |
| 32 | K562/MGIFMNOo | 20p13 | 22310 bp at 5' side: neurensin 2  11871 bp at 3' side: tribbles 3 |
|  | K562/MGIFMNOo | 6p25 | 369512 bp at 5' side: hypothetical protein LOC154386  8891 bp at 3' side: GDP-mannose 4,6-dehydratase |
|  | K562/MGIFMNOo | 1q42 | lin-9 homolog |
|  | K562/MGIFMNOo | 18p11.22 | 178115 bp at 5' side: hypothetical protein XP_002344178  93778 bp at 3' side: NADH dehydrogenase (ubiquinone) flavoprotein 2, 24kDa |
|  | K562/MGIFMNOo | 1q43 | 164 bp at 5' side: hypothetical protein  907 bp at 3' side: galectin-8 isoform b |
|  | K562/MGIFMNOo | 18p11.3 | discs large homolog-associated protein 1 isoform alpha  discs large homolog-associated protein 1 isoform beta |
|  |  |  |  |
| 33 | K562/MGIFMNOo | 2p23.3 | 12938 bp at 5' side: ATPase family, AAA domain containing 2B  18445 bp at 3' side: UBX domain containing 4 |
|  | K562/MGIFMNOo | Multiple | unplaced contig ref\|NT_167216.1 matching several chromosome ends ~5kb from chomosome ends - in several chromosomes |
|  | K562/MGIFMNOo | 6q13 | stromal membrane-associated GTPase-activating protein 1 i... (small ArfGAP) |
|  | K562/MGIFMNOo | 6p22.1 | 44342 bp at 5' side: zinc finger protein 389  15319 bp at 3' side: zinc finger protein 193 |
|  | K562/MGIFMNOo | 20q11.2 | 1596 bp at 5' side: hypothetical protein LOC140688  226938 bp at 3' side: COMM domain containing 7 isoform 1 Disrupts C20orf112, 36kb from ASXL1 (neg orientation) |
|  | K562/MGIFMNOo | 18p11.2 | 13008 bp at 5' side: RAB31, member RAS oncogene family  13492 bp at 3' side: thioredoxin domain-containing 2 isoform 2 |
|  | K562/MGIFMNOo | 1p36.23 | 22368 bp at 5' side: tumor necrosis factor receptor superfamily, member 9 prec...  272 bp at 3' side: Parkinson disease protein 7 |
|  | K562/MGIFMNOo | 11p15 | potassium voltage-gated channel, KQT-like subfamily (KCNQ1) |
|  |  |  |  |
| 34 | K562/MGIFMNOo | 13q32 | transmembrane 9 superfamily member 2 (TM9SF2) |
|  | K562/MGIFMNOo | 2q33 | phospholipase C-like 1 isoform a (PLCL1) |
|  | K562/MGIFMNOo | 2p13 | 2921 bp at 5' side: annexin IV  1228 bp at 3' side: hypothetical protein XP_002342249 |
|  | K562/MGIFMNOo | 5q35.3 | regulator of G-protein signalling 14 |
|  | K562/MGIFMNOo | 1p35.3 | 22092 bp at 5' side: opioid receptor, delta 1  1200 bp at 3' side: erythrocyte membrane protein band 4.1 (elliptocytosis 1, … |
|  | K562/MGIFMNOo | 16p13.3 | 1741 bp at 5' side: hypothetical protein XP_002343484  5730 bp at 3' side: p120E4F |
|  | K562/MGIFMNOo | 7q32 | staphylococcal nuclease domain containing 1 (SND1), ~90kb to mir593, ~150kb to mir129-1 |
|  | K562/MGIFMNOo | 1p34.2 | 9682 bp at 5' side: coiled-coil domain containing 23  Disrupts erythroblast membrane-associated protein precursor (ERMAP) |
|  | K562/MGIFMNOo | 1q42 | polypeptide N-acetylgalactosaminyltransferase 2 (GALNT2) |
|  |  |  |  |
| 35 | K562/MGIFMNOo | 19q12 | no close features Disrupts BC068609 (in 19q12 according to BLAT) |
|  | K562/MGIFMNOo | 8q21.2 | 101663 bp at 5' side: hypothetical protein XP_002342903  154404 bp at 3' side: protein serine kinase H2  Disrupts BF667196 |
|  | K562/MGIFMNOo | 2p12 | 66723 bp at 5' side: hexokinase 2  791 bp at 3' side: DNA polymerase epsilon subunit 4 (POLE4) |
|  | K562/MGIFMNOo | 11p15.4 | 2139 bp at 5' side: hypothetical protein XP_002343087  11141 bp at 3' side: epsilon globin |
|  |  |  |  |
| 36 | K562/MGIFMNOo | 7p13 | 56693 bp at 5' side: hypothetical protein XP_002342760  1282198 bp at 3' side: tensin 3 |
|  | K562/MGIFMNOo | Multiple | unplaced contig ref\|NT_167221.1 matching several chromosome ends |
|  | K562/MGIFMNOo | 8p23 | 497951 bp at 5' side: hypothetical protein XP_002342854  205099 bp at 3' side: CUB and Sushi multiple domains 1 |
|  | K562/MGIFMNOo | Multiple | unplaced contig ref\|NT_167221.1 matching several chromosome ends |
|  | K562/MGIFMNOo | 1p36.2 | 5,10-methylenetetrahydrofolate reductase (NADPH) |
|  | K562/MGIFMNOo | 4q24 | mannosidase, beta A, lysosomal ~70kb at 3’ end NFKB1 |
|  | K562/MGIFMNOo | 17q11.2 | RAB11 family interacting protein 4 (class II)  67kb to NF1 (opposite direction)  105kb to mir193A  121kb to mir365-2 |
|  | K562/MGIFMNOo | 5q33.1 | 10262 bp at 5' side: dynactin 4 (p62) isoform c  11312 bp at 3' side: putative small membrane protein NID67  67kb to RBM22 (possible upregulation) |
|  | K562/MGIFMNOo | 2p13.3 | 2921 bp at 5' side: annexin IV  1227 bp at 3' side: hypothetical protein XP_002342249 65kb to SNRNP27 (possible upregulation) 85kb to MXD1 (possible upregulation) |
|  | K562/MGIFMNOo | 7q32 | staphylococcal nuclease domain containing 1 ~90kb to mir593, ~150kb to mir129-1 |
|  |  |  |  |
