# Supplementary material for: DLGAP1 directs megakaryocytic growth and differentiation in an MPL dependent manner in hematopoietic cells
Source: Biomark Res. 2019 Jul 8;7:13. doi: 10.1186/s40364-019-0165-z (PMC6615210; doi:10.1186/s40364-019-0165-z)
Supplement: Supplementary file 3 — A sample of expression and copy number data on DLGAP1 in hematologic malignancies from Oncomine Platform (www.oncomine.org). (A-D) Expression data. (E-F) Copy number data. (PPTX 93 kb) [file 40364_2019_165_MOESM3_ESM.pptx]

## Slide 1
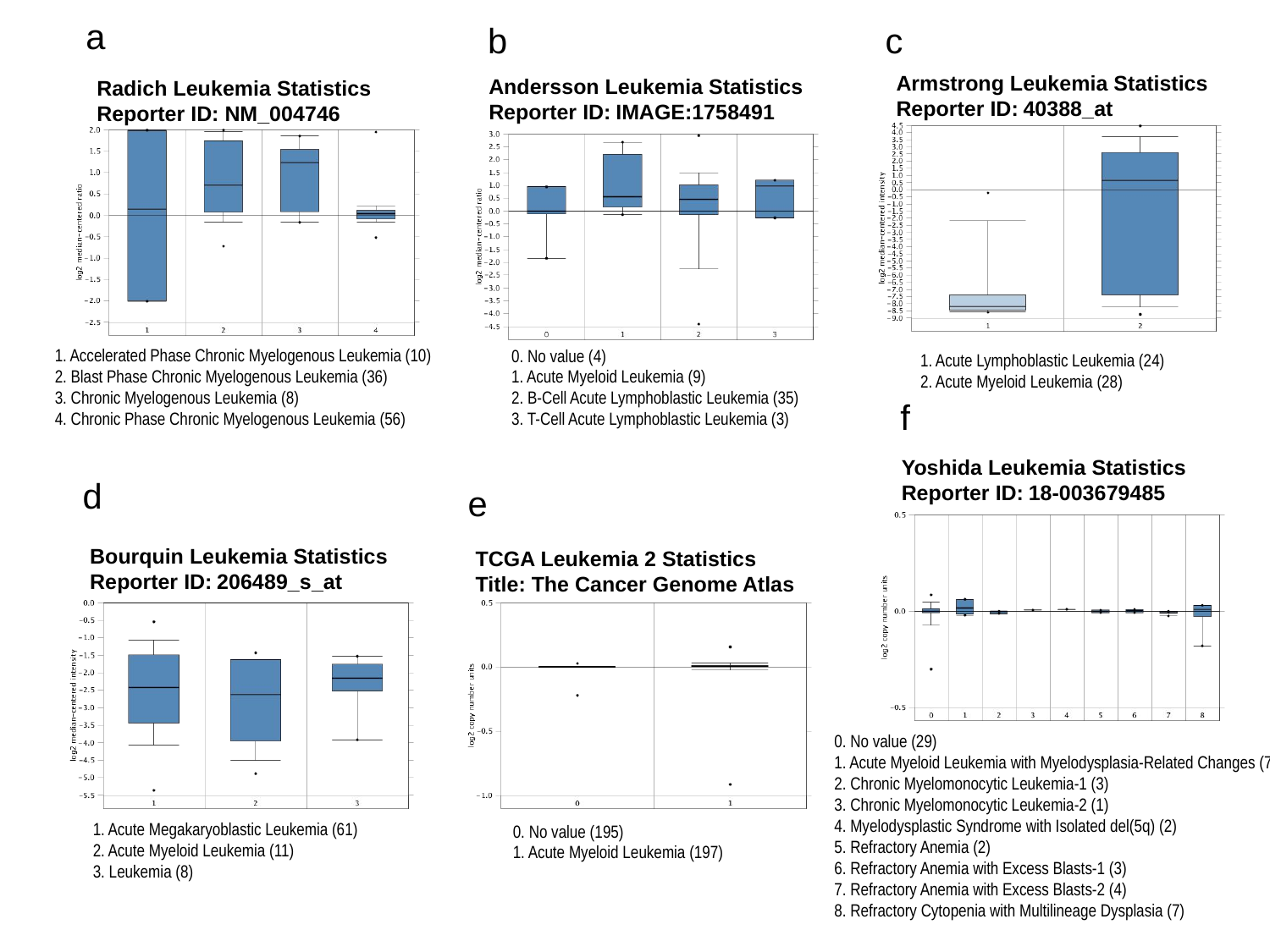

a
b
c
Armstrong Leukemia Statistics
Reporter ID:	40388_at
Andersson Leukemia Statistics
Reporter ID:	IMAGE:1758491
Radich Leukemia Statistics
Reporter ID: NM_004746
1. Accelerated Phase Chronic Myelogenous Leukemia (10)
2. Blast Phase Chronic Myelogenous Leukemia (36)
3. Chronic Myelogenous Leukemia (8)
4. Chronic Phase Chronic Myelogenous Leukemia (56)
0. No value (4)
1. Acute Myeloid Leukemia (9)
2. B-Cell Acute Lymphoblastic Leukemia (35)
3. T-Cell Acute Lymphoblastic Leukemia (3)
1. Acute Lymphoblastic Leukemia (24)
2. Acute Myeloid Leukemia (28)
f
Yoshida Leukemia Statistics
Reporter ID:	18-003679485
d
e
Bourquin Leukemia Statistics
Reporter ID:	206489_s_at
TCGA Leukemia 2 Statistics
Title: The Cancer Genome Atlas
0. No value (29)
1. Acute Myeloid Leukemia with Myelodysplasia-Related Changes (7)
2. Chronic Myelomonocytic Leukemia-1 (3)
3. Chronic Myelomonocytic Leukemia-2 (1)
4. Myelodysplastic Syndrome with Isolated del(5q) (2)
5. Refractory Anemia (2)
6. Refractory Anemia with Excess Blasts-1 (3)
7. Refractory Anemia with Excess Blasts-2 (4)
8. Refractory Cytopenia with Multilineage Dysplasia (7)
1. Acute Megakaryoblastic Leukemia (61)
2. Acute Myeloid Leukemia (11)
3. Leukemia (8)
0. No value (195)
1. Acute Myeloid Leukemia (197)
