# Supplementary material for: DLGAP1 directs megakaryocytic growth and differentiation in an MPL dependent manner in hematopoietic cells
Source: Biomark Res. 2019 Jul 8;7:13. doi: 10.1186/s40364-019-0165-z (PMC6615210; doi:10.1186/s40364-019-0165-z)
Supplement: Supplementary file 4 — (A) Motif consensus sequences and localization of phosphorylation sites in human DLGAP1 protein for hematopoietic relevant Tyrosine kinases. (B) Motif consensus sequences and localization of phosphorylation sites in human DLGAP1 protein for selected hematopoietic relevant Serine kinases. (DOCX 20 kb) [file 40364_2019_165_MOESM4_ESM.docx]

2 A

Tyrosine kinase / phosphatase motifs

| \|  \| \|  \| **Position in query protein** \| **Sequence in query protein** \| **Corresponding motif described in the literature (phosphorylated residues in red)** \| **Features of motif described in the literature** \| **Link to original article describing the motif** \| \| --- \| --- \| --- \| --- \| --- \| --- \| \| 1 \| 43 - 46 \| DHPY \| [E/D]XXpY \| ALK kinase substrate motif \| [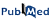](http://www.ncbi.nlm.nih.gov/entrez/query.fcgi?cmd=Retrieve&db=pubmed&dopt=Abstract&list_uids=16273072) \| \| 2 \| 46 - 47 \| YY \| [E/D/Y]pY \| TC-PTP phosphatase substrate motif \| [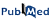](http://www.ncbi.nlm.nih.gov/entrez/query.fcgi?cmd=Retrieve&db=pubmed&dopt=Abstract&list_uids=11352902,12237455) \| \| 3 \| 47 - 48 \| YT \| pY[A/G/S/T/E/D] \| Src kinase substrate motif \| [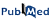](http://www.ncbi.nlm.nih.gov/entrez/query.fcgi?cmd=Retrieve&db=pubmed&dopt=Abstract&list_uids=16273072) \| \| 4 \| 75 - 76 \| YT \| pY[A/G/S/T/E/D] \| Src kinase substrate motif \| [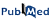](http://www.ncbi.nlm.nih.gov/entrez/query.fcgi?cmd=Retrieve&db=pubmed&dopt=Abstract&list_uids=16273072) \| \| 5 \| 116 - 118 \| DGY \| [E/D]XpY \| SHP1 phosphatase substrate motif \| [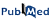](http://www.ncbi.nlm.nih.gov/entrez/query.fcgi?cmd=Retrieve&db=pubmed&dopt=Abstract&list_uids=11994017) \| \| 6 \| 118 - 121 \| YHTL \| pYXX[L/I/V] \| JAK2 kinase substrate motif \| [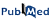](http://www.ncbi.nlm.nih.gov/entrez/query.fcgi?cmd=Retrieve&db=pubmed&dopt=Abstract&list_uids=15143187) \| \| 7 \| 118 - 123 \| YHTLQY \| pYXXXX[F/Y] \| ALK kinase substrate motif \| [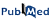](http://www.ncbi.nlm.nih.gov/entrez/query.fcgi?cmd=Retrieve&db=pubmed&dopt=Abstract&list_uids=16273072) \| \| 8 \| 178 - 179 \| YG \| pY[A/G/S/T/E/D] \| Src kinase substrate motif \| [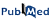](http://www.ncbi.nlm.nih.gov/entrez/query.fcgi?cmd=Retrieve&db=pubmed&dopt=Abstract&list_uids=16273072) \| \| 9 \| 235 - 240 \| YFLEAY \| pYXXXX[F/Y] \| ALK kinase substrate motif \| [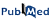](http://www.ncbi.nlm.nih.gov/entrez/query.fcgi?cmd=Retrieve&db=pubmed&dopt=Abstract&list_uids=16273072) \| \| 10 \| 238 - 240 \| EAY \| [E/D]XpY \| SHP1 phosphatase substrate motif \| [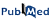](http://www.ncbi.nlm.nih.gov/entrez/query.fcgi?cmd=Retrieve&db=pubmed&dopt=Abstract&list_uids=11994017) \| \| 11 \| 240 - 243 \| YNTI \| pYXX[L/I/V] \| JAK2 kinase substrate motif \| [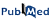](http://www.ncbi.nlm.nih.gov/entrez/query.fcgi?cmd=Retrieve&db=pubmed&dopt=Abstract&list_uids=15143187) \| \| 12 \| 290 - 292 \| EVY \| [E/D]XpY \| SHP1 phosphatase substrate motif \| [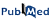](http://www.ncbi.nlm.nih.gov/entrez/query.fcgi?cmd=Retrieve&db=pubmed&dopt=Abstract&list_uids=11994017) \| \| 13 \| 317 - 320 \| YLQV \| pYXX[L/I/V] \| JAK2 kinase substrate motif \| [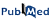](http://www.ncbi.nlm.nih.gov/entrez/query.fcgi?cmd=Retrieve&db=pubmed&dopt=Abstract&list_uids=15143187) \| \| 14 \| 328 - 329 \| YT \| pY[A/G/S/T/E/D] \| Src kinase substrate motif \| [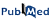](http://www.ncbi.nlm.nih.gov/entrez/query.fcgi?cmd=Retrieve&db=pubmed&dopt=Abstract&list_uids=16273072) \| \| 15 \| 374 - 376 \| ESY \| [E/D]XpY \| SHP1 phosphatase substrate motif \| [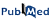](http://www.ncbi.nlm.nih.gov/entrez/query.fcgi?cmd=Retrieve&db=pubmed&dopt=Abstract&list_uids=11994017) \| \| 16 \| 439 - 441 \| ESY \| [E/D]XpY \| SHP1 phosphatase substrate motif \| [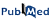](http://www.ncbi.nlm.nih.gov/entrez/query.fcgi?cmd=Retrieve&db=pubmed&dopt=Abstract&list_uids=11994017) \| \| 17 \| 568 - 570 \| DAY \| [E/D]XpY \| SHP1 phosphatase substrate motif \| [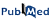](http://www.ncbi.nlm.nih.gov/entrez/query.fcgi?cmd=Retrieve&db=pubmed&dopt=Abstract&list_uids=11994017) \| \| 18 \| 967 - 969 \| EIY \| [E/D]XpY \| SHP1 phosphatase substrate motif \| [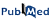](http://www.ncbi.nlm.nih.gov/entrez/query.fcgi?cmd=Retrieve&db=pubmed&dopt=Abstract&list_uids=11994017) \| \|  \| \| --- \| --- \| --- \| --- \| --- \| --- \| --- \| --- \| --- \| --- \| --- \| --- \| --- \| --- \| --- \| --- \| --- \| --- \| --- \| --- \| --- \| --- \| --- \| --- \| --- \| --- \| --- \| --- \| --- \| --- \| --- \| --- \| --- \| --- \| --- \| --- \| --- \| --- \| --- \| --- \| --- \| --- \| --- \| --- \| --- \| --- \| --- \| --- \| --- \| --- \| --- \| --- \| --- \| --- \| --- \| --- \| --- \| --- \| --- \| --- \| --- \| --- \| --- \| --- \| --- \| --- \| --- \| --- \| --- \| --- \| --- \| --- \| --- \| --- \| --- \| --- \| --- \| --- \| --- \| --- \| --- \| --- \| --- \| --- \| --- \| --- \| --- \| --- \| --- \| --- \| --- \| --- \| --- \| --- \| --- \| --- \| --- \| --- \| --- \| --- \| --- \| --- \| --- \| --- \| --- \| --- \| --- \| --- \| --- \| --- \| --- \| --- \| --- \| --- \| --- \| --- \| --- \| \|  \| \| \| |
| --- | --- | --- | --- | --- | --- | --- | --- | --- | --- | --- | --- | --- | --- | --- | --- | --- | --- | --- | --- | --- | --- | --- | --- | --- | --- | --- | --- | --- | --- | --- | --- | --- | --- | --- | --- | --- | --- | --- | --- | --- | --- | --- | --- | --- | --- | --- | --- | --- | --- | --- | --- | --- | --- | --- | --- | --- | --- | --- | --- | --- | --- | --- | --- | --- | --- | --- | --- | --- | --- | --- | --- | --- | --- | --- | --- | --- | --- | --- | --- | --- | --- | --- | --- | --- | --- | --- | --- | --- | --- | --- | --- | --- | --- | --- | --- | --- | --- | --- | --- | --- | --- | --- | --- | --- | --- | --- | --- | --- | --- | --- | --- | --- | --- | --- | --- | --- | --- | --- | --- | --- |

2 B

Selected Serine kinase / phosphatase motifs

| **Position in query protein** | **Sequence in query protein** | **Corresponding motif described in the literature (phosphorylated residues in red)** | **Features of motif described in the literature** | **Link to original article describing the motif** |
| --- | --- | --- | --- | --- |
| Multiple | SACDS | pSXXX[pS/pT] | MAPKAPK2 kinase substrate motif | [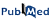](http://www.ncbi.nlm.nih.gov/entrez/query.fcgi?cmd=Retrieve&db=pubmed&dopt=Abstract&list_uids=15629715,8280084) |
| Multiple | LLSP | XXpSP | GSK-3, ERK1, ERK2, CDK5 substrate motif | [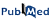](http://www.ncbi.nlm.nih.gov/entrez/query.fcgi?cmd=Retrieve&db=pubmed&dopt=Abstract&list_uids=16020478) |
| Multiple | LSP | X[pS/pT]P | GSK-3, ERK1, ERK2, CDK5 substrate motif | [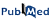](http://www.ncbi.nlm.nih.gov/entrez/query.fcgi?cmd=Retrieve&db=pubmed&dopt=Abstract&list_uids=16020478) |
| Multiple | SPGR | [pS/pT]PX[R/K] | CDK1, 2, 4, 6 kinase substrate motif | [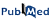](http://www.ncbi.nlm.nih.gov/entrez/query.fcgi?cmd=Retrieve&db=pubmed&dopt=Abstract&list_uids=12501191,10607671) |
| Multiple | SPGRI | pSPX[R/K]X | CDK kinase substrate motif | [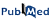](http://www.ncbi.nlm.nih.gov/entrez/query.fcgi?cmd=Retrieve&db=pubmed&dopt=Abstract&list_uids=16273072) |
| Multiple | RMRSGS | [R/K]XRXXpS | MAPKAPK1 kinase substrate motif | [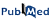](http://www.ncbi.nlm.nih.gov/entrez/query.fcgi?cmd=Retrieve&db=pubmed&dopt=Abstract&list_uids=7498520) |
